# CircRNF144B/miR-342-3p/FBXL11 axis reduced autophagy and promoted the progression of ovarian cancer by increasing the ubiquitination of Beclin-1

**DOI:** 10.1038/s41419-022-05286-7

**Published:** 2022-10-08

**Authors:** Wenting Song, Zhirui Zeng, Yilei Zhang, Haili Li, Huimin Cheng, Jun Wang, Fengrui Wu

**Affiliations:** 1 Anhui Province Key Laboratory of Environmental Hormone and Reproduction, Fuyang Normal University, Fuyang, 236037 Anhui China; 2 Anhui Province Key Laboratory of Embryo Development and Reproductive Regulation, Fuyang Normal University, Fuyang, 236037 Anhui China; 3 Basic medical school, Guizhou Medical University, Guiyang, 550025 Guizhou China; 4 Reproductive medicine center, Guizhou Medical University, Guiyang, 550025 Guizhou China

**Keywords:** Tumour biomarkers, Metastasis

## Abstract

Circular RNAs (circRNAs) can regulate autophagy and ovarian cancer (OC) progression. However, autophagy-associated circRNAs involved in OC progression are largely unknown. Bioinformatics, RNA sequencing, and qRT-PCR were conducted to detect circRNF144B expression in OC as well as its relationship with patient prognosis. Functional experiments were used to determine the effects of circRNF144B on the proliferation, mobility and autophagy of OC. Double luciferase reporter assays, immunoprecipitation, and ubiquitination detection were performed to determine the molecular mechanisms of circRNF144B in autophagy and OC progression. CircRNF144B was elevated in OC tissues with low autophagy levels, and associated with poor prognosis. CircRNF144B promoted the malignant biological properties of OC cells, and inhibited the autophagy. Mechanistically, circRNF144B acts as a sponge for miR-342-3p and inhibits miR-342-3p-induced degradation of lysine demethylase 2 A (FBXL11) mRNA, leading to elevated FBXL11 protein levels. Elevated FBXL11 promoted the ubiquitination and degradation of Beclin-1, thus inhibiting autophagy. In conclusion, CircRNF144B increased FBXL11 level by sponging miR-342-3p, whereas elevated FBXL11 promoted the ubiquitination and protein degradation of Beclin-1, thus suppressing autophagy flux and promoting OC progression. Thus, circRNF144B may be an effective target for OC therapy.

## Introduction

Ovarian cancer (OC) is a common malignancy in women, with high morbidity and mortality [1, 2]. Surgery combined with chemotherapy and radiotherapy remains the primary therapeutic strategy for OC. However, to date, the 5-year survival rate of patients with OC is still <40% towing to a high rate of recurrence and metastasis [3, 4]. Therefore, uncovering the molecular mechanisms involved in OC progression may contribute to the development of OC therapy.

Circular RNAs (CircRNAs) are a class of novel non-coding RNAs that do not contain a 5′ -cap and 3′ -poly(A) tail and form a circular structure by covalent bonding [5, 6]. The biological functions of circRNAs have been widely explored in several diseases, including autoimmune diseases [7], diabetes mellitus, and cancer [8]. Previous studies have indicated that circRNAs can sponge miRNAs and suppress miRNA-target gene degradation induced by miRNAs [9]. Dysregulation of circRNAs has been shown to promote OC progression. CircPLEKHM3 is reduced in OC cells, thus accelerating the activation of the AKT pathway and OC progression [10]. CircWHSC1 sponges miR-145 and miR-1182 and increases the expression of the oncogene mucin 1 and telomerase reverse transcriptase, leading to accelerated proliferation and metastasis [11]. The elevated level of circ0007841 was observed in OC tissues, and OC tissues with high circ0007841 levels had a shorter overall survival rate [12]. However, circRNAs involved in OC progression remain largely unknown.

Autophagy is widely expressed in eukaryotic cells under physiological and pathological conditions. Autophagy not only maintains intracellular homeostasis but also provides nutrients for cell survival by decomposing damaged organelles and proteins [13, 14]. The effects of autophagy on OC are complex Beclin 1 is a remarkable regulator of autophagy initiation and is involved in autophagosome formation. A total of 40–75% of OC tissues demonstrated monoallelic deletion of the gene that encodes Beclin; therefore, reduced expression of Beclin-1 and lower autophagy levels were observed in OC tissues in comparison to adjacent tissues [15]. Lower Beclin-1 expression was positively related to advanced FIGO stages in OC. Moreover, the expression of LC3, a biomarker for autophagy, was lower in OC tissues from patients in stages III-IV in comparison to those from patients in stages I-II [16]. Furthermore, some therapeutics have been developed to induce excessive autophagy in OC cells, thereby causing apoptosis [17, 18]. This evidence indicates that autophagy deficiency may contribute to OC tumorigenesis and progression. However, a series of studies has indicated that autophagy is elevated in OC cells under stress conditions, including hypoxia and chemotherapy, and assists OC cells to survive [19, 20]. These conflicting studies suggest that autophagy may act as an adverse factor under certain circumstances. However, until now, autophagy-associated circRNAs as well as their effects in regulating autophagy and OC progression are mostly unknown.

The current study was aimed to identify key autophagy-associated circRNAs in OC and explore their effects and molecular mechanisms in the autophagy and progression of OC. It was demonstrated that circRNF144B is a key autophagy-associated circRNA that is elevated in OC tissues with low autophagy levels and predicts poor prognosis in OC. CircRNF144B sponged miR-342-3p and increased the expression of FBXL11. Elevated FBXL11 induced by circRNF144B promoted the ubiquitination of Beclin-1 and its protein degradation, leading to autophagy deficiency and OC progression. CircRNF144B can act as a prominent target for OC therapy.

## Materials and methods

### Clinical specimen

A total of 36 OC and adjacent non-tumor tissues were obtained in the Affiliated Hospital of Guizhou Medical University (Guizhou, China) and approved by the Human Ethics Committee of Guizhou Medical University. Samples were quickly frozen at −80 °C after surgery until the experiments were performed. None of the patients had received radiotherapy or chemotherapy before sample collection. All patients provided written informed consent prior to clinical specimen collection.

### Bioinformatics analysis for the autophagy signature in OC tissues in The Cancer Genome Atlas (TCGA)

First, we downloaded the RNA-seq data and overall survival rate (OS) information of 373 patients with OC from TCGA (https://portal.gdc.cancer.gov). Before conducting the follow-up analysis, RNA-seq data were merged, normalized, and annotated. A defined set of autophagy genes was downloaded from GSEA (http://amigo.geneontology.org/amigo/term/GO:0010508). The expression of autophagy genes was extracted and used to calculate the autophagy signature of each sample via the t-distributed stochastic neighbor embedding (t-SNE) method. According to their autophagy signature and Euclidean distance calculated based on autophagy gene expression, OC tissues were then clustered into high (*n* = 128) and low autophagy groups (*n* = 245).

### Immunohistochemical staining (IHC)

IHC was performed on OC and adjacent tissues. After deparaffinized with xylene and rehydrated with a gradient of ethanol to distilled water, blocked the section with goat serum. The sections were then incubated with a primary antibody against FBXL11 (1:500; Cat No. 24311-1-AP, Proteintech, Wuhan, China), labeled with an avidin-biotin-peroxidase. The stained results were reviewed and scored as the staining extent and positive range.

### Immunofluorescence

OC tissues were immobilized using 4% paraformaldehyde and permeabilized via 0.5% Triton (Beyotime, Jiangsu, China). After washing three times with PBS, 5% BSA was used to block the tissues. OC tissues were then incubated with an anti-LC3 primary antibody (1:50; Cat No. 14600-1-AP, Proteintech, Wuhan, China) at 4 °C overnight. Then, a FITC conjunct-second antibody was incubated with OC tissues for 2 h, and DAPI was used to label cell nuclei. Finally, a 90i fluorescence microscope (Nikon, Japan) was used to obtain the fluorescence signal of the LC3 dots in each OC tissue.

### RNA-sequencing

RNA sequencing was performed to identify circRNAs between OC tissues with high LC3 dots (*n* = 3) and low dots (*n* = 3) as follows: total RNA in tissues was acquired by a TRIzol reagent (Sangon Biotech Co., Ltd.; Shanghai, China) based on the manufacturer’s instructions. Ribosomal RNA and linear RNA were removed using the MGIEasy rRNA Removal Kit (MGI, Shenzhen, China) and RNase R (Thermo Fisher Scientific, USA), respectively. Fragmentation buffer (Ambion, USA) was used to break the residual RNA into short fragments, which acted as templates to synthesize cDNA. A QiaQuick PCR Extraction Kit (Sigma-Aldrich, USA) was utilized to purify double-stranded cDNAs, which were then dissolved in EB. Following agarose gel electrophoresis, suitable fragments were used to perform PCR-amplifying in order to construct a cDNA library. The prepared cDNA library was then sequenced on an Illumina HiSeq 2500 platform by SeqHealth Biotechnology Co. (Wuhan, China). Differentially expressed circRNAs were analyzed in R software by the EdgeR package. P < 0.05, and |LogFC | >1 were set as the cut-off to identify differentially expressed circRNAs.

### qRT-PCR

The mRNA levels of circRNAs, miRNAs, and mRNAs in OC tissues or cells were determined by qRT-PCR method. Briefly, total RNA in OC tissues or cells was extracted using a TRIzol reagent (Sangon Biotech Co., Ltd.; Shanghai, China). The SuperScript IV Reverse Transcription Kit (Thermo Fisher Scientific, USA) was used to transform circRNAs and mRNAs into cDNA, while the highly sensitive M-MuLV Reverse Transcriptase (Sangon Biotech Co., Ltd; Shanghai, China) was conducted to synthesize cDNA for miRNA. RT-qPCR amplification was conducted using SYBR reagent (Roche, USA) on a LightCycler 96 instrument (Roche, USA). GAPDH was set as a reference for circRNAs and mRNAs, whereas U6 was set as the loading control for miRNAs. The primers used are listed in Supplementary Table 1.

### Cell culture and transfection

The ovarian epithelial cell line (IOSE80) and OC cell lines (A2780, HEY-T30, SKOV3, OVCAR-3, ES2, and OV-1063) were obtained from the American Type Culture Collection (USA). OC cells were cultured in DMEM containing 10% FBS at 37 °C, whereas IOSE80 cells were cultured in RPMI-1640 medium containing 10% FBS at 37 °C. Short hairpin RNAs (shRNAs) targeting circRNF144B, FBXL11, and their negative control (NC) were acquired from GeneChem (Shanghai, China) and subcloned into pSuper-retro-puro. MiR-342-3p mimic, inhibitor, and NC were also acquired from Genechem. Small interfering RNAs (siRNAs) targeting FBXL11 and NC were accessed from HANBIO (Shanghai, China). We constructed pCMV5-circRNF144B, pCMV5-HA-FBXL11, and pCMV5-flag-Beclin-1 to overexpress circRNF144B, HA-FBXL11, and Beclin-1. Polybrene reagent (Sangon Biotech Co., Ltd; Shanghai, China) was used to perform transfection. After transfection with shRNAs for 48 h, OC cells were selected for 10 days under puromycin treatment to obtain stably low-expressing cells; OC cells were selected for 14 days under geneticin reagent (G418) treatment to obtain high-expressing cells after transfection with plasmids for 48 h.

### Actinomycin D assay

First, 2 mg/mL actinomycin D (Thermo Fisher Scientific, USA) was used to suppress transcription in OC cells. Then, after 0, 4, 8, 12, 16, and 20 h, total RNA in OC cells was extracted. Finally, the expression of linerRNF144B and circRNF144B in the RNA products was then determined by qRT-PCR method.

### RNase R treatment assays

Total 4 μg of RNA was extracted from OC cells by a TRIzol reagent and equally divided as treatment and control groups. RNase R (4 U/μg) was added to the RNA in the treatment group, whereas the solvent was used to treat the RNA in the control group. Finally, the products were analyzed using RT-qPCR.

### Nuclear-cytoplasmic fractionation

Cytoplasmic & Nuclear RNA Purification Kit (Norgen, USA) was conducted to extract nuclear and cytoplasmic RNA according to the manufacturer’s instructions. The proportion of circRNF144B in the nucleus and cytoplasm was determined by a qRT-PCR method, whereas GAPDH and U6 were used as nuclear and cytoplasmic references.

### Fluorescence in situ hybridization (FISH)

The Cy3-conjugated circRNF144B probes were constructed and obtained from Genechem Co., Ltd. and used for detecting the sub-localization of circRNF144B in OC cells. The OC cells were fixed in the coverslips by 4% paraformaldehyde, and the cells were digested by protease K (Thermo Fisher Scientific). Gradient alcohol (70, 85, and 100%) was then used to dehydrate the cells. Followed by diluting in hybridization solution and degeneration, the Cy3-conjuncted circRNF144B probes were added in OC cells overnight at 40 °C under a dark environment. Finally, formamide, NP-40, and DAPI were added to the OC cells, and circRNF144B signals in OC tissues were detected using a confocal microscope (Olympus, Japan).

### Methods for detection of cell proliferation in vitro

The CCK-8, colony formation, and 5-ethynyl-2’-deoxyuridine absorb assays were performed to detect OC cell proliferation in vitro. CCK-8 reagent (Yeasen, Shanghai, China) and BeyoClick™ EdU-488 kit were used to perform CCK-8 and 5-Ethynyl-2 absorb assays, respectively, according to the manufacturer’s instructions. For colony formation assay, 1000 OC cells were set in a 6-well plate and cultured at 37 °C for 14 days. The medium was then removed, and 4% paraformaldehyde was used to fix the cell colony. Finally, the cell colonies were stained with crystal violet, and colony number in each well was calculated.

### Methods for detection of cell mobility in vitro

The migration and invasion of OC cells were detected by wound healing and transwell assays, respectively. For the wound healing assay, OC cells were set in 6-well plates and cultured until the confluence >95%. Then, a 200 μL-tip was utilized to create a wound in the cell monolayer. The wound conditions at 0 and 24 h were recorded to calculate the area of cell migration. For the transwell assay, 2 × 10^5^ OC cells resuspended in 200 μL DMEM without FBS were placed in the upper chamber (Corning, USA) pre-coated with 7% Matrigel (Millipore, USA). Then, 700 μL of DMEM containing 10% FBS was added to the lower chamber as an inductive substance. After 24 h, 4% paraformaldehyde and 0.1% crystal violet solution were used to fix and stain the upper chamber, respectively. After washing the residual crystal violet, an orthotopic light microscope (Olympus, Japan) was used for collecting the field of the upper chamber, and the invasive cell number in each field was calculated using the ImageJ software.

### In vivo experiments

The in vivo experiments were approved by the Animal Research Ethical Committee of Guizhou Medical University. For the subcutaneous tumorigenesis model, a total of 2 × 10^6^ SKOV3 cells with circRNF144B-inhibition, circRNF144B-overexpression, and NC-treatment were injected into the left flank of mice (*n* = 5 for each group). The health condition of the mice was observed daily and the tissue volume was recorded weekly. After feeding for 30 days, the mice were euthanized via cervical dislocation method, and tumor tissues were extracted to photo and weight. To detect cell metastasis, 2 × 10^6^ SKOV3 cells with circRNF144B-inhibition, circRNF144B-overexpression, and NC-treatment were injected into the caudal vein of mice. The health condition of the mice was recorded daily, and the mice were euthanized by cervical dislocation method after 4-weeks feeding. Finally, the lungs were extracted and HE staining was performed.

### Western blotting

Total protein in OC cells was extracted by a potent RIPA lysis reagent (Millipore, USA) containing 1% PMSF (Millipore, USA). The protein concentration of each sample was determined using the BCA method, and then samples (30 μg) were subjected to electrophoresis on an SDS-PAGE gel (Mellon, Dalian, China) and transferred onto PVDF membranes (Merck, USA). To prevent non-specific signals, the membranes were incubated with 5% BSA (Merck, USA) for 2 h. The primary antibodies, including LC3 (1:500; Cat No. 14600-1-AP, Proteintech, Wuhan, China), P62 (1:1000; Cat No. 18420-1-AP, Proteintech, Wuhan, China), FBXL11 (1:500; Cat No. 24311-1-AP, Proteintech, Wuhan, China), Beclin-1 (1:500; Cat No. 11306-1-AP, Proteintech, Wuhan, China), HA (1:5000; Cat No. 51064-2-AP, Proteintech, Wuhan, China), Flag (1:1000; Cat No. 80010-1-RR, Proteintech, Wuhan, China), and GAPDH (1:2000; Cat No. 60004-1-Ig, Proteintech, Wuhan, China) were added to incubate PVDF membranes at 4 °C overnight. After washing TBST for thrice, secondary antibodies were incubated for 2 h, and the protein signals in membranes were detected using the ECL reagent (Yeasen, Shanghai, China). GAPDH was set as a loading control to calculate the expression level of the target proteins.

### Detection of mRFP-GFP-LC3 dot

Adenovirus for overexpressing mRFP-GFP-LC3 was obtained from HanBio (Wuhan, China) and transfected into OC cells via polybrene. After transfection for 48 h, cells were set into confocal dish. Follow by adding treatment factors, OC cells were fixed with 4% paraformaldehyde and detected in confocal microscopy (Olympus, Japan). Yellow dots in OC cells indicated the autophagosomes, while red dots indicated autolysosomes.

### Dual-luciferase reporter assay

The binding relationship among circRNF144B, miR-342-3p, and FBXL11 was analyzed using a dual-luciferase reporter system (Promega Corporation), according to the manufacturer’s instructions. Wild-type or mutant reporters of circRNF144B and FBXL11 were constructed and transfected into OC cells which were pre-transfected with NC mimic or miR-342-3p mimic, respectively. The fluorescence intensity of each group of cells was determined using a Modulus fluorescence detector (TurnerBioSystems, USA).

### Immunoprecipitation

Total protein in OC cells was extracted by a mild RIPA lysis reagent (Thermo Fisher Scientific, USA) containing 1% PMSF and 1% phosphorylase inhibitor (Boster, Wuhan, China). Primary anti-FBXL11 (1 μg), anti-HA (1 μg; Cat No. 51064-2-AP, Proteintech, Wuhan, China), and anti-Flag antibodies (1 μg; Cat No. 80010-1-RR, Proteintech, Wuhan, China) were used to bind FBXL11, HA-conjunct, and Flag-conjunct proteins at 4 °C overnight. Agarose bead gel (Beyotime, Suzhou, China) was used to enrich the antigen-antibody complex at 4 °C for 3 h. Then, after washing PBS for thrice, agarose beads were obtained using centrifugation. The agarose bead gel was mixed with 2× loading buffer and performed degeneration at 100 °C for 10 min. Proteins interacting with FBXL11 were identified via LC/MS and visualized via silver staining using a rapid silver stain kit (Beyotime, Suzhou, China) according to the manufacturer’s instructions. Finally, the expression of target proteins was verified by western blotting.

### Statistical analysis

All experiments were performed three times. Data are presented as mean ± standard deviation and were analyzed using SPSS 19.0. An independent t-test was conducted to analyze the data derived from the two groups, while one-way analysis of variance followed by Bonferroni’s host test was used to analyze data derived from multiple groups. The co-expression relationship between circRNF144B, miR-342-3p, and FBXL11 in OC tissues was analyzed using Pearson’s correlation analysis. The difference in the overall survival rate between the two groups was analyzed via Kaplan–Meier survival analysis. Statistical significance was set at *P* < 0.05.

## Results

### CircRNF144B was elevated in OC tissues and predicted a poor prognosis

**Fig. 1:**
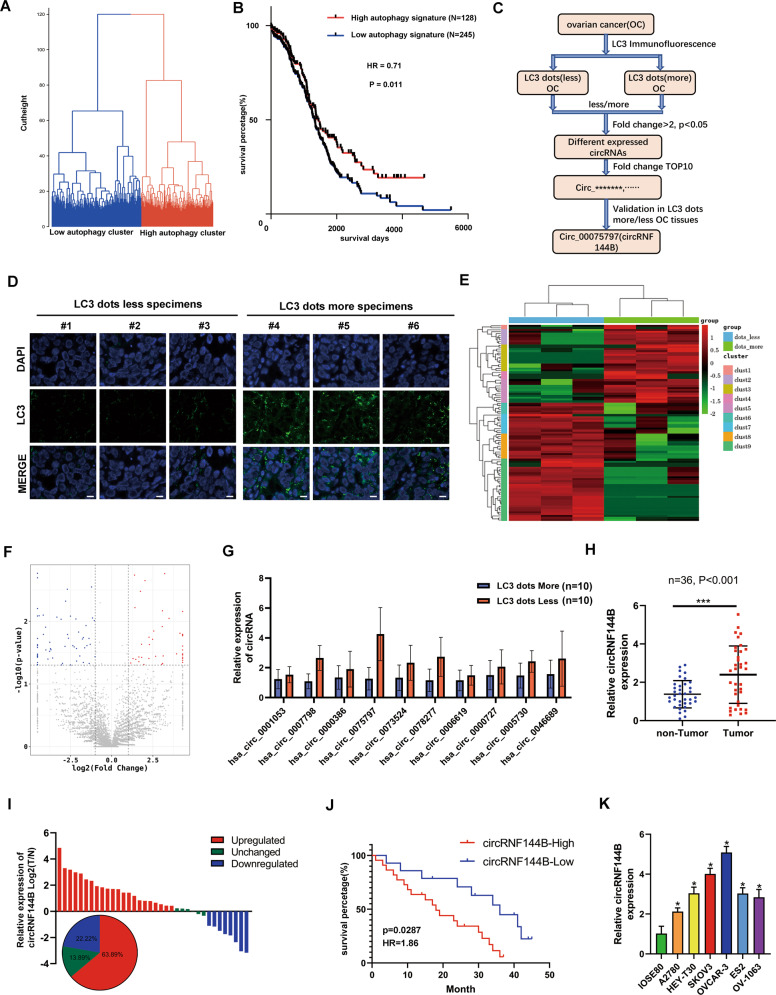
CircRNF144B was increased in OC tissues and predicted poor outcome. **A** OC tissues in the TCGA database were clustered into high autophagy signature and low autophagy signature groups according to their autophagy signature score. **B** The overall survival rate of patients with OC in the high autophagy signature and low autophagy signature groups. **C** Flow chart of key autophagy-associated circRNAs screening in OC. **D** Tissue fluorescence indicated the LC3 dots in OC tissues. Scale bar, 25 μm. **E** Heatmap indicated the upregulated and downregulated circRNAs between OC tissues with high LC dots and low LC3 dots. **F** Volcano plot indicated the gene change between OC tissues with high LC dots and low LC3 dots. **G** qRT-PCR was used to detect the expression of top 10 upregulated-circRNAs in OC tissues with high LC3 dots and low LC3 dots. **H** qRT-PCR was used to detect the expression of circRNF144B in OC tissues and adjacent tissues. **I** Differential expression of circRNF144B between OC tissues and adjacent tissues in patients based on the results detected by qRT-PCR. **J** Survival rate between patients with high and low circRNF144B expression. **K** qRT-PCR was used to detect the expression of circRNF144B in OC cells (A2780, HEY-T30, SKOV3, OVCAR-3, ES2, and OV-1063) and normal ovarian epithelial cell line IOSE80. *, *P* < 0.05; ***, *P* < 0.001.

By calculating the autophagy signature using the t-SNE method, OC tissues in TCGA were clustered into high-and low-autophagy signature groups (Fig. 1A). A shorter overall survival rate was observed in OC tissues in the low autophagy signature group (Fig. 1B). This evidence suggests that autophagy may be a suppressor of OC. Therefore, we developed a strategy to explore the autophagy-associated circRNAs (Fig. 1C). OC tissues with high and low LC3 dots (*n* = 3 per group) were used for RNA sequencing (Fig. 1D). A total of 61 upregulated and 40 downregulated circRNAs were observed in OC tissues with low LC3 dots *vs*. samples with high LC3 dots (Fig. 1E-F). Then, the top 10 upregulated circRNAs expression in another 10 high autophagy tissues and 10 low autophagy tissues samples were detected by qRT-PCR method, and found that the expression of has-circ-0075797 (also named circRNF144B) was most significantly elevated in samples with low LC3 dots (Fig. 1G). Moreover, we indicated that circRNF144B expression was elevated in OC tissues in comparison to that in adjacent nontumor tissues (Fig. 1H). Similarly, circRNF144B was also increased in most OC tissues (63.89%) compared to adjacent non-tumor tissues (Fig. 1I), and patients provided the OC tissues with high circRNF144B levels had lower survival rates (Fig. 1J). Furthermore, the expression of circRNF144B was also elevated in OC cells (A2780, HEY-T30, SKOV3, OVCAR-3, ES2, and OV-1063) in comparison to that in the normal ovarian epithelial cell line IOSE80 (Fig. 1K). Moreover, the clinicopathological characteristic analysis found that circRNF144B-High was associated with lymph node metastasis in OC patients (Supplementary Table 2). In conclusion, circRNF144B was found to be a key autophagy-associated circRNA involved in OC progression.

### CircRNF144B was originated from RNF144B and mostly distributed in the cytoplasm

**Fig. 2:**
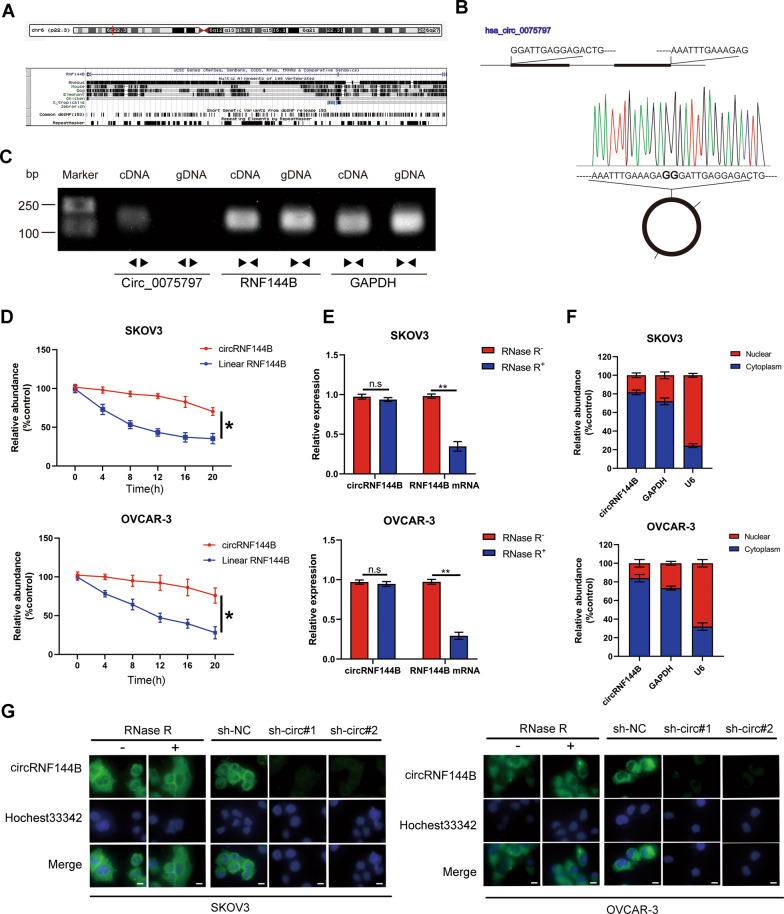
CircRNF144B was verified as a cytoplasmic circRNA. **A** CircRNA annotation in the circBase database indicated that circRNF144B is located on chromosome 6, contains 367 bp, and derives from the host gene RNF144B. **B** Sanger sequencing verified the head-to-tail splicing of circRNF144B. **C** Nucleic acid electrophoresis indicated that circRNF144B was amplified by divergent primers in the cDNA, but not in the gDNA. **D** qRT-PCR results indicated that the mRNA stability of circRNF144B was higher than that of linear RNF144B. **E** qRT-PCR results indicated that circRNF144B exhibited resistance to RNase. **F**–**G** Nuclear-cytoplasm separation and FISH assays indicated that circRNF144B was located in the cytoplasm. Scale bar, 25 μm. **, *P* < 0.01.

circRNA annotation performed in the circBase database demonstrated that circRNF144B is located on chromosome 6 (18399729-18439975), contains 367 bp in length, and is derived from the host gene RNF144B (Fig. 2A). Sanger sequencing further verified the head-to-tail splicing of circRNF144B (Fig. 2B). Similarly, cDNA and gDNA extracted from SKOV3 cells were used for nucleic acid electrophoresis, and the evidences indicated that circRNF144B was amplified by divergent primers in cDNA but not in gDNA (Fig. 2C). Actinomycin was used to inhibit RNA synthesis in SKOV3 and OVCAR-3; the degradation rate of circRNF144B was lower than that of linear RNF144B (Fig. 2D). Similarly, circRNF144B exhibited resistance to RNase when compared to its host gene (Fig. 2E). Moreover, circRNF144B was mostly located in the cytoplasm of SKOV3 and OVCAR-3 cell (Fig. 2F-G).

### circRNF144B promoted OC cell progression and reduced autophagy

**Fig. 3:**
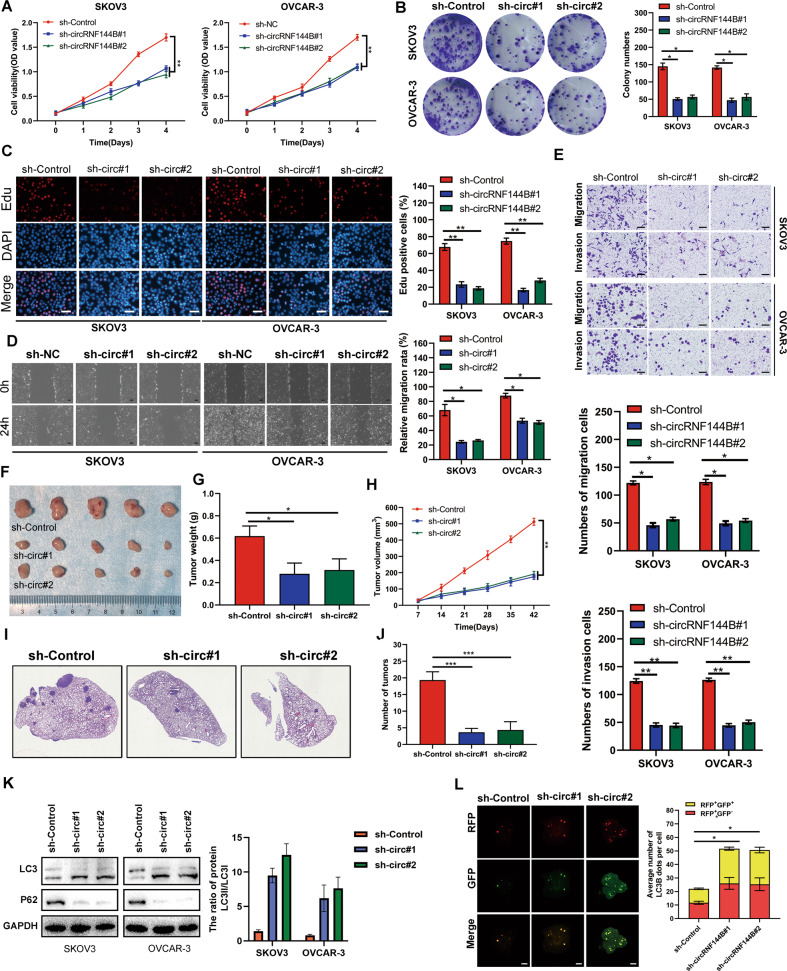
Inhibition of circRNF144B reduced OC cell proliferation and mobility, as well as increased autophagy. **A** CCK-8 results indicated that circRNF144B knockdown inhibited the cell viability of SKOV3 and OVCAR-3. **B** Colony formation assay indicated that circRNF144B knockdown inhibited cell colony formation in SKOV3 and OVCAR-3 cells. **C** EDU positive rate was reduced in SKOV3 and OVCAR-3 cells with circRNF144B knockdown. Scale bar, 100 μm. **D** Wound healing assay was used to detect the effects of circRNF144B knockdown on SKOV3 and OVCAR-3 cell migration. Scale bar, 50 μm. **E** Transwell assay was used to detect the effects of circRNF144B knockdown on SKOV3 and OVCAR-3 cell invasion. Scale bar, 50 μm. **F**–**H** The volume and weight of tumor tissues derived from SKOV3 cells with circRNF144B knockdown and control SKOV3 cells. **I-J** Number of metastatic foci in the lung tissues from the mice injected with SKOV3 cells with circRNF144B knockdown and control SKOV3 cells. **K** Western blot was used to detect the expression of LC3 and P62 in SKOV3 and OVCAR-3 cells after circRNF144B knockdown. **L** Confocal microscopy was used to detect the number of autophagosomes and autophagolysosomes in SKOV3 cell after circRNF144B knockdown. Scale bar, 25 μm. *, *P* < 0.05; **, *P* < 0.01; ***, P < 0.001.

Targeting circRNF144B shRNA was carried out to suppress circRNF144B-expression in SKOV3 and OVCAR-3 cells (Figure S1A). CCK-8 (Fig. 3A) and colony formation assays (Fig. 3B) indicated that suppression of circRNF144B reduced the cell viability and colony formation of SKOV3 and OVCAR-3 cells. SKOV3 and OVCAR-3 cells with circRNF144B-inhibition had a lower EDU positive rate (Fig. 3C). Suppression of circRNF144B obviously decreased the mobility of SKOV3 and OVCAR-3 cells in vitro (Fig. 3D-E). Moreover, it was demonstrated that tumor tissues derived from SKOV3 cells with circRNF144B knockdown grew slowly in vivo and had lower weights (Fig. 3F-H). The results of the pulmonary metastasis model indicated that SKOV3 cells with circRNF144B knockdown exhibited lower metastatic ability than control cells in vivo (Fig. 3I-J). Furthermore, SKOV3 and OVCAR-3 cells with circRNF144B knockdown had higher LC3 expression and lower P62 expression than control cells (Fig. 3K), as well as increased autophagic vacuoles (Fig. 3L, Figure S2). Consistent with the above results, we found that overexpression of circFBXL11 significantly upregulated the proliferation and migration ability of ovarian cancer cells and decreased the level of autophagy (Figs. S3,4). These results indicate that circRNF144B promoted OC cell progression and reduced autophagy.

### miR-342-3p was sponged by circRNF144B and played as a suppressor in OC cells

**Fig. 4:**
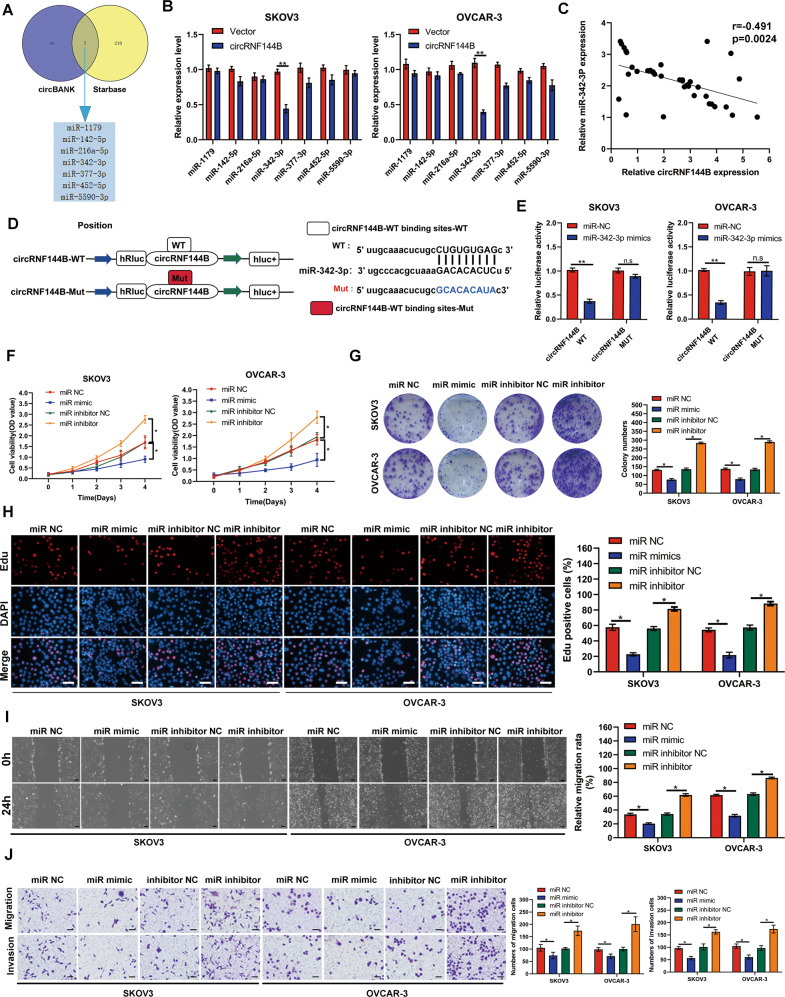
MiR-342-3p was identified as a target miRNA of circRNF144B, and played as a suppressor in OC. **A** Two online databases including CircBank and Starbase were used to predict the target miRNAs of circRNF144B. **B** Expression of seven potential target miRNAs was detected by qRT-PCR in SKOV3 and OVCAR-3 cells with circRNF144B-overexpression. **C** Pearson correlation analysis for circRNF144B and miR-342-3p in OC tissues. **D** The binding site between circRNF144B and miR-342-3p. **E** Double luciferase reporting assays indicated the direct binding between circRNF144B and miR-342-3p. **F** CCK-8 was used to determine the cell viability of SKOV3 and OVCAR-3 cells after miR-342-3p-knockdown and miR-342-3p-overexpression. **G** Colony formation assay was used to determine colony formation in SKOV3 and OVCAR-3 cells after miR-342-3p-knockdown and miR-342-3p-overexpression. **H** EDU-positive rates were detected in SKOV3 and OVCAR-3 cells after miR-342-3p-knockdown and miR-342-3p-overexpression. Scale bar, 100 μm. **I–J** Wound healing and transwell assays were used to detect the migration and invasion of SKOV3 and OVCAR-3 cells after miR-342-3p-knockdown and miR-342-3p-overexpression. Scale bar, 50 μm.*, *P* < 0.05; **, *P* < 0.01.

Using two databases including CircBank and Starbase, total seven miRNAs including miR-1179, miR-142-5p, miR-216a-5p, miR-342-3p, miR-377-3p, miR-452-5p and miR-5590-3p were predicted as target miRNAs of circRNF144B (Fig. 4A). Among them, miR-342-3p was most obviously reduced in SKOV3 and OVCAR-3 cells with circRNF144B-overexpression (Fig. 4B). Interestingly, miR-342-3p expression was negatively associated with circRNF144B expression in OC tissues (Fig. 4C). Based on the binding site between miR-342-3p and circRNF144B, circRNF144B dual-fluorescence reporter plasmids with wild-type and mutant binding sites were constructed (Fig. 4D). It was demonstrated that miR-342-3p significantly decreased the fluorescence intensity in SKOV3 and OVCAR-3 cells transfected with circRNF144B dual-fluorescence reporter plasmids containing the wild-type binding site, but not in those containing the mutant binding site (Fig. 4E). Therefore, miR-342-3p was considered a target miRNA of circRNF144B in OC. Moreover, evidences revealed that miR-342-3p-overexpression reduced the viability and colony formation of SKOV3 and OVCAR-3 cells, whereas the inhibition of miR-342-3p induced the opposite effects (Fig. 4F-G). EDU assays indicated that miR-342-3p-overexpression reduced the EDU-positive rate, whereas suppression of miR-342-3p elevated the EDU-positive rate (Fig. 4H). Furthermore, overexpression of miR-342-3p inhibited the mobility of SKOV3 and OVCAR-3 cells, while the inhibition of miR-342-3p promoted cell migration and invasion (Fig. 4I-J). This evidence indicates that miR-342-3p, a target circRNF144B, acts as a suppressor in OC.

### FBXL11 was determined as a target gene of miR-342-3p and played as an oncogene in OC

**Fig. 5:**
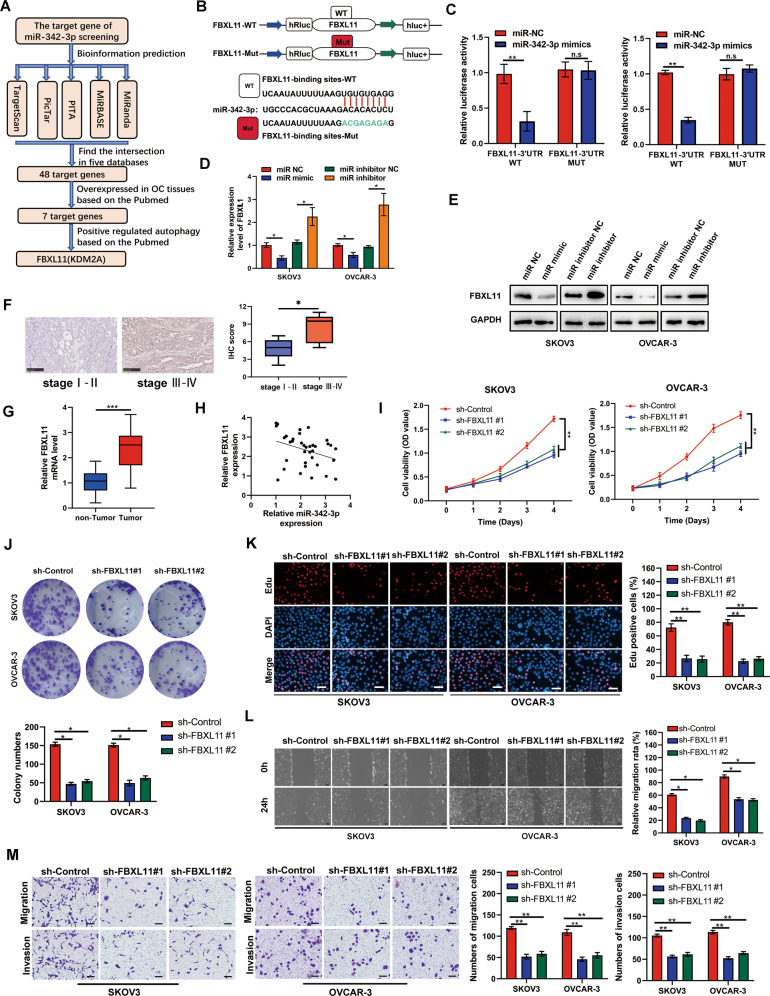
FBXL11 was identified as a target mRNA of miR-342-3p, and played as an oncogene in OC. **A** FBXL11 was determined as the target mRNA of miR-342-3p according to the flow chart. **B** Binding site between miR-342-3p and FBXL11 was exhibited. **C** Double luciferase reporting assays indicated the direct binding between FBXL11 and miR-342-3p. **D** qRT-PCR was used to detect the mRNA levels of FBXL11 in SKOV3 and OVCAR-3 cells after miR-342-3p-knockdown and miR-342-3p-overexpression. **E** Western blot was used to detect the protein levels of FBXL11 in SKOV3 and OVCAR-3 cells after miR-342-3p-knockdown and miR-342-3p-overexpression. **F-G** IHC was used to detect the expression levels of FBXL11 in OC tissues with different stage. **G** qRT-PCR were used to detect the expression levels of FBXL11 in OC tissues and adjacent tissues. **H** Pearson correlation analysis for FBXL11 and miR-342-3p in OC tissues. **I** CCK-8 assay was used to determine viability of SKOV3 and OVCAR-3 cells after FBXL11 knockdown. **J** Colony formation assay was used to detect colony formation in SKOV3 and OVCAR-3 cells after FBXL11 knockdown. **K** EDU positive rate in SKOV3 and OVCAR-3 cells after FBXL11 knockdown was detected. Scale bar, 100 μm. **L-M** Wound healing assay and transwell assay was used to detect the migration and invasion of SKOV3 and OVCAR-3 cells after FBXL11 knockdown. Scale bar, 50 μm. *, *P* < 0.05; **, *P* < 0.01.

The target genes of miR-342-3p were explored according to the flow diagram, and through bioinformatics analysis in five prediction databases, a total of 48 genes were predicted, while 7 of them were found to be elevated in OC tissues according to previous studies; among them, FBXL11 has shown its potential to regulate autophagy in previous studies (Fig. 5A). Then, we found that FBXL11 siRNAs could significantly inhibit FBXL11 expression, siRNA#1 was selected for following experiment (Fig. S5). Therefore, we investigated whether FBXL11 is a key target gene of miR-342-3p. We transfected FBXL11 dual-fluorescence reporter plasmids with wild-type and mutant binding sites into SKOV3 and OVCAR-3 cells (Fig. 5B). MiR-342-3p reduced the fluorescence intensity in SKOV3 and OVCAR-3 cells transfected with FBXL11 dual-fluorescence reporter plasmids containing wild-type binding sites, but not in those containing mutant binding sites (Fig. 5C). MiR-342-3p-overexpression decreased the mRNA and protein levels of FBXL11, whereas suppression of miR-342-3p increased their expression (Fig. 5D-E). Furthermore, FBXLL11 expression level was elevated in OC tissues, positively with OC stage (Fig. 5F-G) and negatively co-expressed with miR-342-3p (Fig. 5H). This indicates that FBXL11 was a key target gene of miR-342-3p. The CCK-8 results indicated that FBXL11 knockdown reduced cell viability (Fig. 5I). Colony formation assay indicated that colony formation ability in SKOV3 and OVCAR-3 cells with FBXL11 inhibition was obviously reduced (Fig. 5J). EDU assays indicated that FBXL11-knockdown group cells had a lower EDU-positive rate (Fig. 5K). Knockdown of FBXL11 reduced cell migration and invasion (Fig. 5L-M). These results indicate that FBXL11, as a target gene of miR-342-3p, may act as an oncogene in OC.

### The effects of circRNF144B were dependent on miR-342-3p/FBXL11 axis

**Fig. 6:**
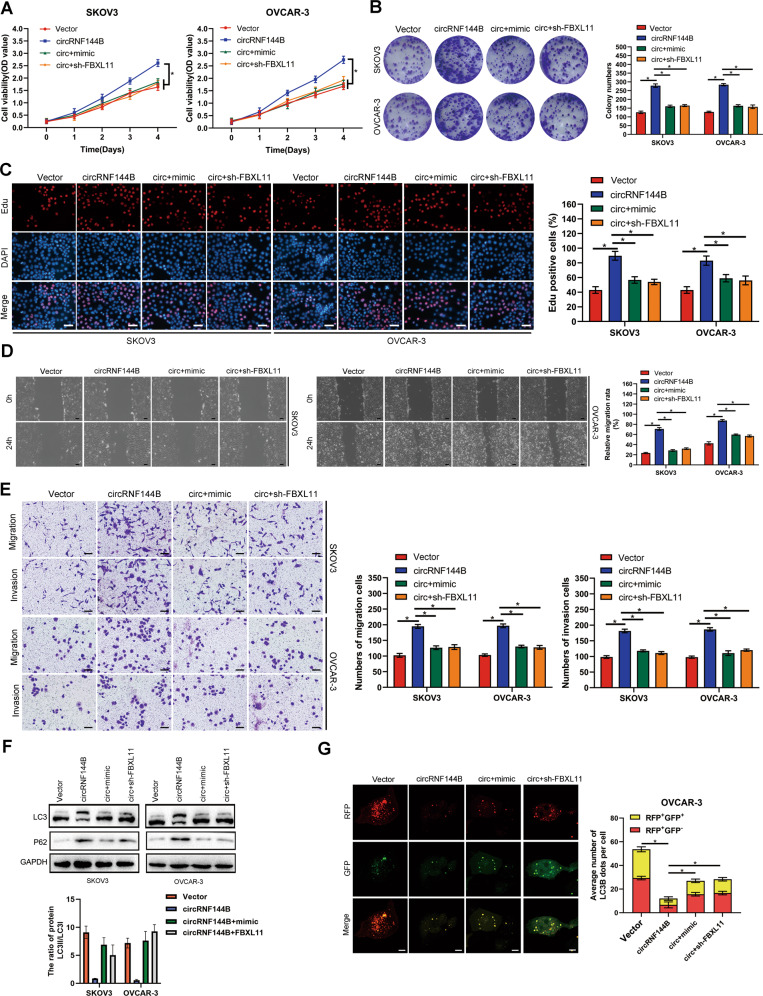
Effects of circRNF144B was dependent on miR-342-3p/FBXL11 axis. MiR-342-3p was overexpressed, and FBXL11 was suppressed in SKOV3 and OVCAR-3 cells. **A** The CCK-8 assay was used to detect cell viability in each group. **B** Colony formation assay was used to detect colony formation in each group. **C** EDU positive rate in each group was detected. Scale bar, 100 μm. **D** Wound healing assay was used to detect cell migration in each group. Scale bar, 50 μm. **E** Transwell assay was used to detect cell invasion in each group. Scale bar, 50 μm. **F** Western blotting was used to detect LC3 and P62 in each group. **G** Confocal microscopy was used to detect the number of autophagosomes and autophagolysosomes in each group. Scale bar, 25 μm. *, *P* < 0.05; **, *P* < 0.01.

We then determined whether the miR-342-3p/FBXL11 axis was involved in the biological functions of circRNF144B in OC cell progression and autophagy. CCK-8 and colony formation assays indicated that both miR-342-3p-overexpression and FBXL11-inhibition relieved the effects of circRNF144B on cell viability and colony formation (Fig. 6A-B). Similarly, EDU assays indicated that miR-342-3p-overexpression and FBXL11-inhibition reduced the EDU-positive rate in cells overexpressing circRNF144B (Fig. 6C). Moreover, both miR-342-3p-overexpression and FBXL11-inhibition reversed the stimulatory effects of circRNF144B on SKOV3 and OVCAR-3 cell mobility (Fig. 6D-E). Furthermore, the inhibitory effects of circRNF144B on autophagy in SKOV3 and OVCAR-3 cells were also reversed by miR-342-3p-overexpression or FBXL11-inhibition (Fig. 6F-G, Fig. S6). In conclusion, the effects of circRNF144B on OC cells are dependent on the miR-342-3p/FBXL11 axis.

### FBXL11 enhanced ubiquitin-mediated degradation of Beclin-1

**Fig. 7:**
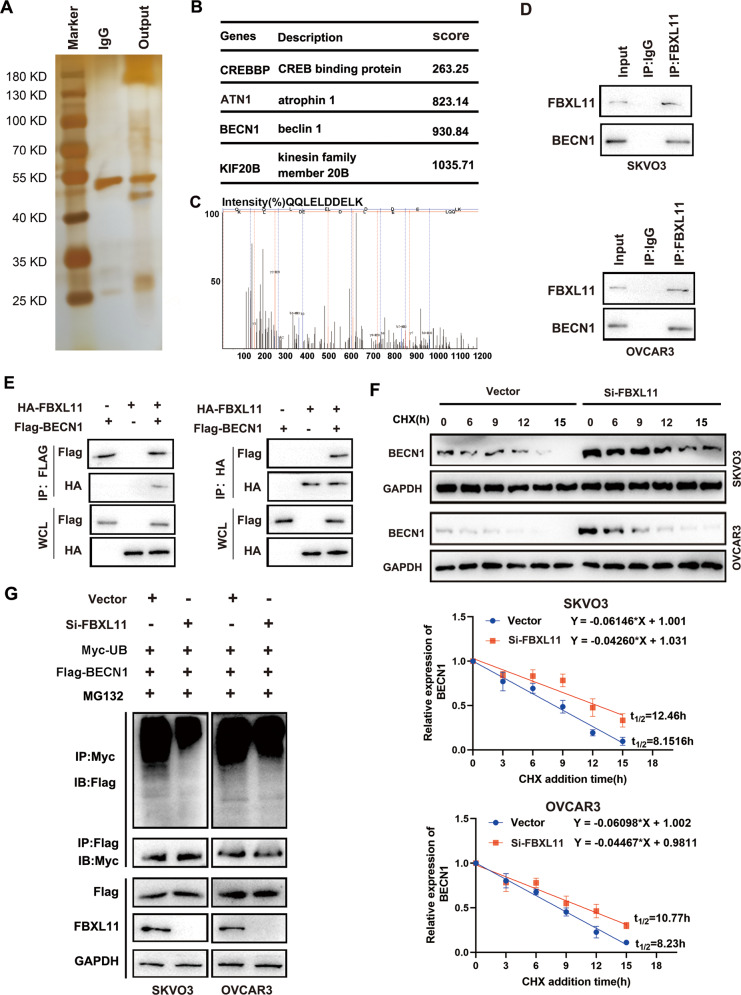
FBXL11 increased the ubiquitination of Beclin-1 in OC cells. **A** Silver staining indicated protein binding with FBXL11. **B** Top 4 interacting proteins with FBXL11 are shown. **C** The secondary mass spectrograms indicated BECN1 binding with FBXL11. **D** Endogenic immunoprecipitation indicated the binding between FBXL11 and Beclin-1. **E** Ectogenic immunoprecipitation indicated the binding between FBXL11 and Beclin-1. **F** CHX was used to inhibit protein synthesis, and degradation of Beclin-1 was detected in SKOV3 and OVCAR-3 cells with FBXL11 knockdown and control. **G** The ubiquitination level of Beclin-1 in SKOV3 and OVCAR-3 cells with FBXL11 knockdown and control was detected.

We then determined the molecular mechanisms of FBXL11 regulation by circRNAs in autophagy. Silver staining was performed to identify the interacting proteins of FBXL11, and four interacting proteins of FBXL11 at high concentrations were found, including CREB binding protein, atrophin 1, Beclin-1, and kinesin family member 20B (Fig. 7A-B). The secondary mass spectrograms indicated these proteins binding with FBXL11(Fig. 7C, Fig. S7). We further determined whether FBXL11 directly regulated Beclin-1. Endogenous and exogenous IP experiments were performed, and the results indicated that FBXL11 was directly bound to Beclin-1 (Fig. 7D-E). Furthermore, the protein degradation rate of Beclin-1 was obviously reduced in SKOV3 and OVCAR-3 cells upon FBXL11-inhibition (Fig. 7F). Moreover, we found that the ubiquitination level of Beclin-1 was reduced in SKOV3 and OVCAR-3 cells upon FBXL11-inhibition (Fig. 7G).

### Rescued autophagy in OC cells reduced the stimulative effects of circRNF144B

**Fig. 8:**
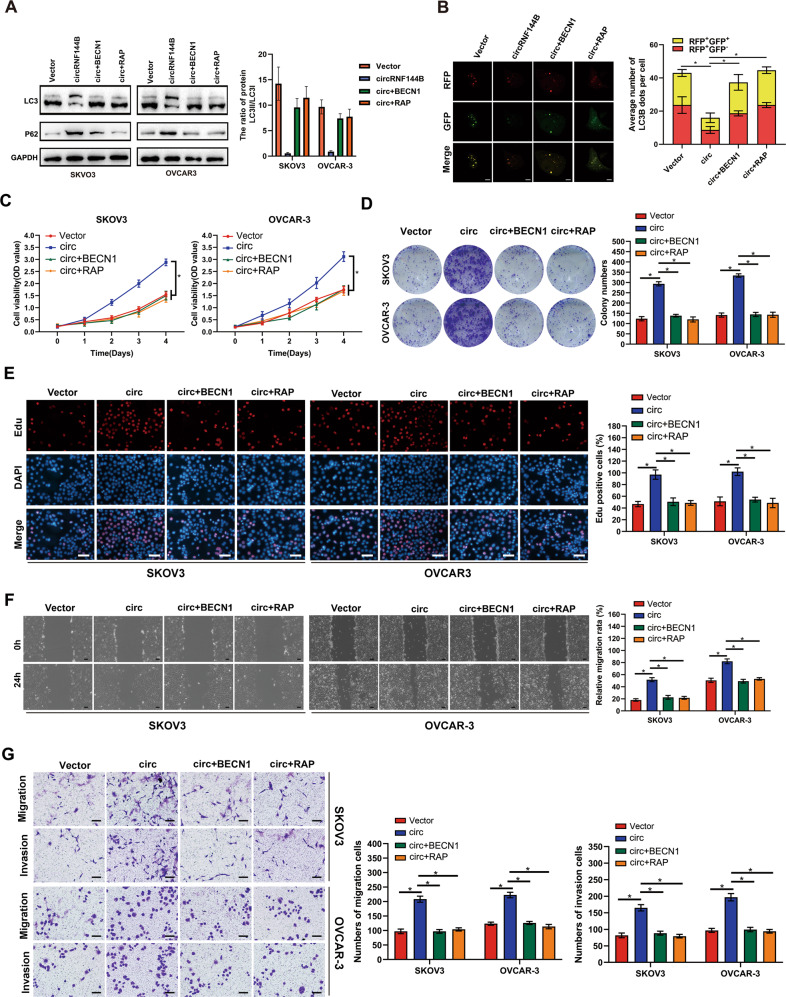
Rescued autophagy in OC cells reversed the effects of circRNF144B. OC cells were divided into four groups according to treatment factors: negative control (NC), circRNF144B-overepression, circRNF144B-overexpression + Beclin-1-overexpression and circRNF144B-overexpression + rapamycin (RAP) treatment. **A** Western blotting was used to detect LC3 and P62 in each group of cells. **B** Confocal microscopy was used to detect the number of autophagosomes and autophagolysosomes in each cell group. Scale bar, 25 μm. **C** CCK-8 was used to detect the cell viability in each group cells. **D** Colony formation assay was used to detect colony formation in each group of cells. **E** EDU positive rate in each group was detected. Scale bar, 100 μm **F** Wound healing assay was used to detect cell migration in each group cells. Scale bar, 50 μm. **G** Transwell assay was used to detect the invasion in each group of cells. Scale bar, 50 μm. *,*P* < 0.05.

We then rescued autophagy in SKOV3 and OVCAR-3 cells with circRNF144B-overexpression via Beclin-1 overexpression and rapamycin treatment (Fig. 8A-B, Fig. S8). Rescue of autophagy in SKOV3 and OVCAR-3 cells with circRNF144B-overexpression significantly reduced cell viability and colony formation (Fig. 8C-D). EDU assays indicated that rescued autophagy in SKOV3 and OVCAR-3 cells with ircRNF144B-overexpression significantly reduced the EDU-positive rate (Fig. 8E). Moreover, the accelerative effects of circRNF144B on SKOV3 and OVCAR-3 cell migration and metastasis were reversed by Beclin-1 overexpression and rapamycin treatment (Fig. 8F-G).

## Discussion

CircRNAs, a class of non-coding RNA, are participated in OC progression by sponging target miRNAs and increasing target mRNA expression [21]. However, to date, the dysregulated circRNAs in OC are largely unknown. In the current study, we demonstrated that the autophagy-related circRNA circRNF144B is upregulated in OC tissues and cell lines. Knockdown of circRNF144B decreased OC cell proliferation and mobility and increased the autophagy level in OC cells, whereas circRNF144B-overexpression induced opposite effects in OC cells. Mechanistically, circRNF144B sponged miR-342-3p and increased the expression of FBXL11 while inducing Beclin-1 ubiquitination, thus inhibiting autophagy.

A series of dysregulated circRNAs involved in OC progression has been reported in previous studies. For example, circ0009910 is upregulated in OC tissues, whereas high expression of circ0009910 predicts poor outcomes [22]. CircCELSR1 is upregulated in OC cells after paclitaxel chemotherapy and increases cell resistance to paclitaxel [23]. High circRNA1656 expression is associated with advanced clinical stages [24]. CircATRNL1 has the potential to activate the Smad4 signaling pathway and exhibited its suppressor functions on inhibiting angiogenesis and OC cell metastasis [25]. In the present study, we provide the first evidence that circRNF144B is an onco-circRNA in OC that is upregulated in OC tissues, predicts poor prognosis, and promotes OC cell proliferation and mobility. CircRNF144B maybe a novel biomarker and target for OC diagnosis and therapy.

It is widely known that dysregulated autophagy is a key mediator in the development of OC. Low autophagy and its biomarkers, including LC3, Beclin-1, and ATG7, have been observed in OC tissues in a series of previous studies, which were also associated with lower overall survival rates and advanced clinical stages [26, 27]. Therefore, autophagy inhibition may be considered a factor that induces the progression of OC. Beclin-1 interacts with BCL-2 and is involved in the process of autophagy by promoting the formation of the VPS34-PIK3CA complex [28, 29]. In most patients with OC, monoallelic deletion of the gene that encodes Beclin-1 is observed and is considered a key factor mediating autophagy inhibition in OC tissues. Similarly, some transcriptional regulatory mechanisms also contribute to Beclin-1 deletion in OC. ALKBH5 acts as an oncogene in OC and decreases the mRNA level of Beclin-1 by upregulating miR-7 [30]. STATs suppresses the transcription of Beclin-1 and promotes OC progression [31]. In the present study, we uncovered a novel post-transcriptional mechanism for Beclin-1 in OC. We demonstrated that FBXL11, a ubiquitinated protein regulated by circRNF144B, exhibited its effects to induce Beclin-1 ubiquitination and inhibited autophagy. Autophagy by Beclin-1-overexpression and rapamycin treatment reduced the effects of circRNF144B. CircRNF144B plays as an onco-circRNA in OC, which promotes OC cell proliferation and mobility by promoting ubiquitin-mediated degradation of Beclin-1 mediated by the miR-342-3p/FBXL11 axis.

## Supplementary information

Supplementary Table 1

Supplementary Table 2

Supplementary Figure

Original Data File

aj-checklist

## Data Availability

The datasets supporting the conclusions of this article are included within the article and its additional files.
