# Supplementary material for: CircRNF144B/miR-342-3p/FBXL11 axis reduced autophagy and promoted the progression of ovarian cancer by increasing the ubiquitination of Beclin-1
Source: Cell Death Dis. 2022 Oct 8;13(10):857. doi: 10.1038/s41419-022-05286-7 (PMC9547922; doi:10.1038/s41419-022-05286-7)
Supplement: Supplementary file 1 — Supplementary Table 1 [file 41419_2022_5286_MOESM1_ESM.doc]

| **Primer** | **Forward (5’-3’)** | **Reverse (5’-3’)** |
| --- | --- | --- |
| circRNF144B | TGTGCCTAAACCACGGGAC | TTTCAGCAGTCATGGCGAGA |
| miRNA-342-3p | GTGCTATCTGTGATTGAGGGA | CGGGTGCGATTTCTGTG |
| U6 | GGAACGATACAGAGAAGATTAGC | TGGAACGCTTCACGAATTTGCG |
| FBXL11 | CCAAAGGTGCGGGTTCCTAC | GGCTCCTGACACAATCGGG |
| GAPDH | ACAACTTTGGTATCGTGGAAGG | GCCATCACGCCACAGTTTC |

**Table 1. qPCR Primers used in the study**
