# Supplementary material for: CircRNF144B/miR-342-3p/FBXL11 axis reduced autophagy and promoted the progression of ovarian cancer by increasing the ubiquitination of Beclin-1
Source: Cell Death Dis. 2022 Oct 8;13(10):857. doi: 10.1038/s41419-022-05286-7 (PMC9547922; doi:10.1038/s41419-022-05286-7)
Supplement: Supplementary file 2 — Supplementary Table 2 [file 41419_2022_5286_MOESM2_ESM.docx]

| **Clinicopathologic Feature** |  | **CircRNF144B** | | ***p value*** |
| --- | --- | --- | --- | --- |
|  |  | **High expression** | **Low expression** |  |
| All cases |  | 19 | 17 |  |
| Age |  |  |  | 0.9999 |
|  | ≤50 | 7 | 7 |  |
|  | ＞50 | 12 | 10 |  |
| FIGO staging |  |  |  | 0.2814 |
|  | I + II | 4 | 7 |  |
|  | III + IV | 15 | 10 |  |
| Lymphatic metastasis |  |  |  | **0.0166** |
|  | Negative | 4 | 11 |  |
|  | Positive | 15 | 6 |  |
| CA125 (U/ml) |  |  |  | 0.4338 |
|  | <600 | 3 | 5 |  |
|  | ≥600 | 16 | 12 |  |

**Table 2. Correlation between circRNF144B expression and clinicopathological characteristics in ovarian cancer**
