# Supplementary material for: CircRNF144B/miR-342-3p/FBXL11 axis reduced autophagy and promoted the progression of ovarian cancer by increasing the ubiquitination of Beclin-1
Source: Cell Death Dis. 2022 Oct 8;13(10):857. doi: 10.1038/s41419-022-05286-7 (PMC9547922; doi:10.1038/s41419-022-05286-7)

Figure S1 A-B q-RT-PCR experiment verified the transfection efficiency of shRNA and overexpressed lenti-virus on CircRNF144B in SKOV3 and OVCAR-3.


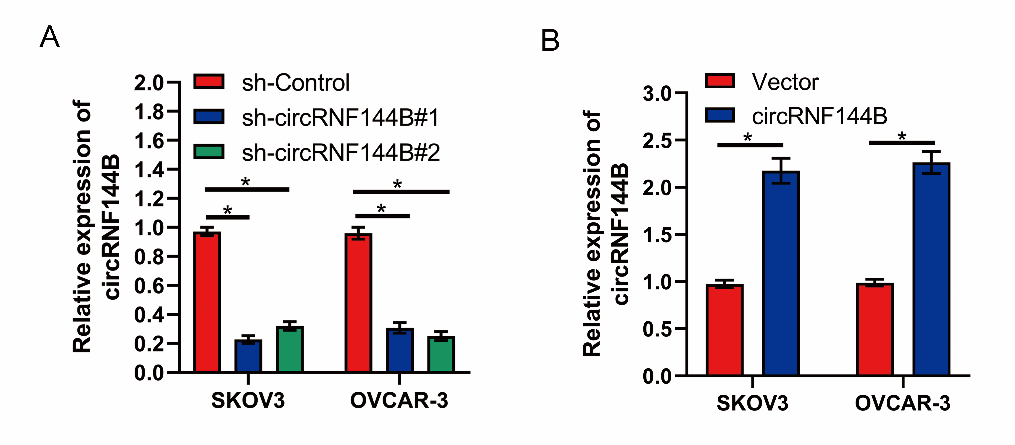


Figure S2 Confocal microscopy was used to detect the number of autophagosomes and autophagolysosomes in OVCAR-3 cells after circRNF144B knockdown.


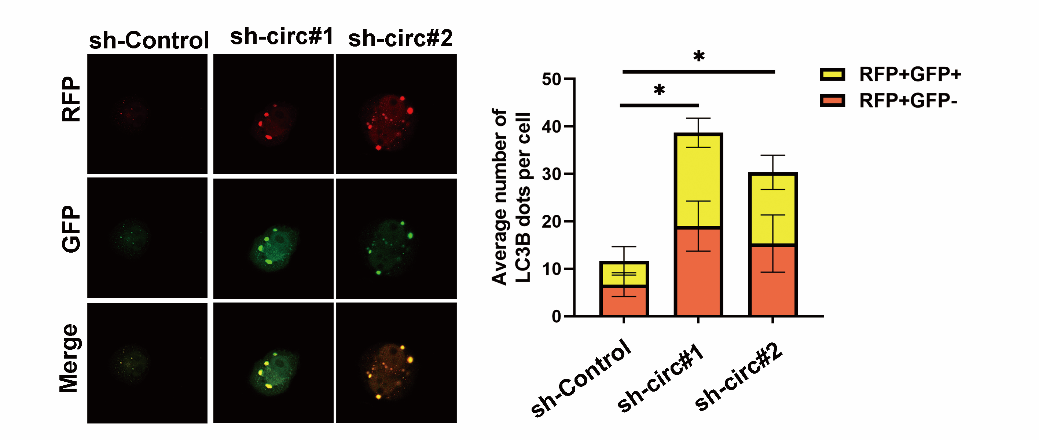


Figure S3 **CircRNF144B-overexpression increased OC cell proliferation and mobility, as well as suppressed autophagy. A** CCK-8 results indicated that circRNF144B-overexpression increased the viability of SKOV3 and OVCAR-3 cells. **B** Colony formation assay indicated that circRNF144B-overexpression enhanced colony formation in SKOV3 and OVCAR-3 cells. **C** EDU positive rate was increased in SKOV3 and OVCAR-3 cells with circRNF144B-overexpression. Scale bar, 100μm. **D** Transwell assay was used to detect the effects of circRNF144B-overexpression on SKOV3 and OVCAR-3 cell invasion. Scale bar, 50μm. **E** Wound healing assay was used to detect the effects of circRNF144B-overexpression on SKOV3 and OVCAR-3 cell migration. Scale bar, 50μm. **F-H** The volume and weight of tumor tissues derived from SKOV3 cells with circRNF144B-overexpression and control SKOV3 cells. **I-J** Number of metastatic foci in the lung tissues from the mice injected with SKOV3 cells with circRNF144B-overexpression and control SKOV3 cells. **K** Western blot was used to detect the expression of LC3 and P62 in SKOV3 and OVCAR-3 cells after circRNF144B-overexpression. **L** Confocal microscopy was used to detect the number of autophagosomes and autophagolysosomes in SKOV3 cells after circRNF144B-overexpression. Scale bar, 25μm.*, P<0.05; **, P<0.01; ***, P<0.001.


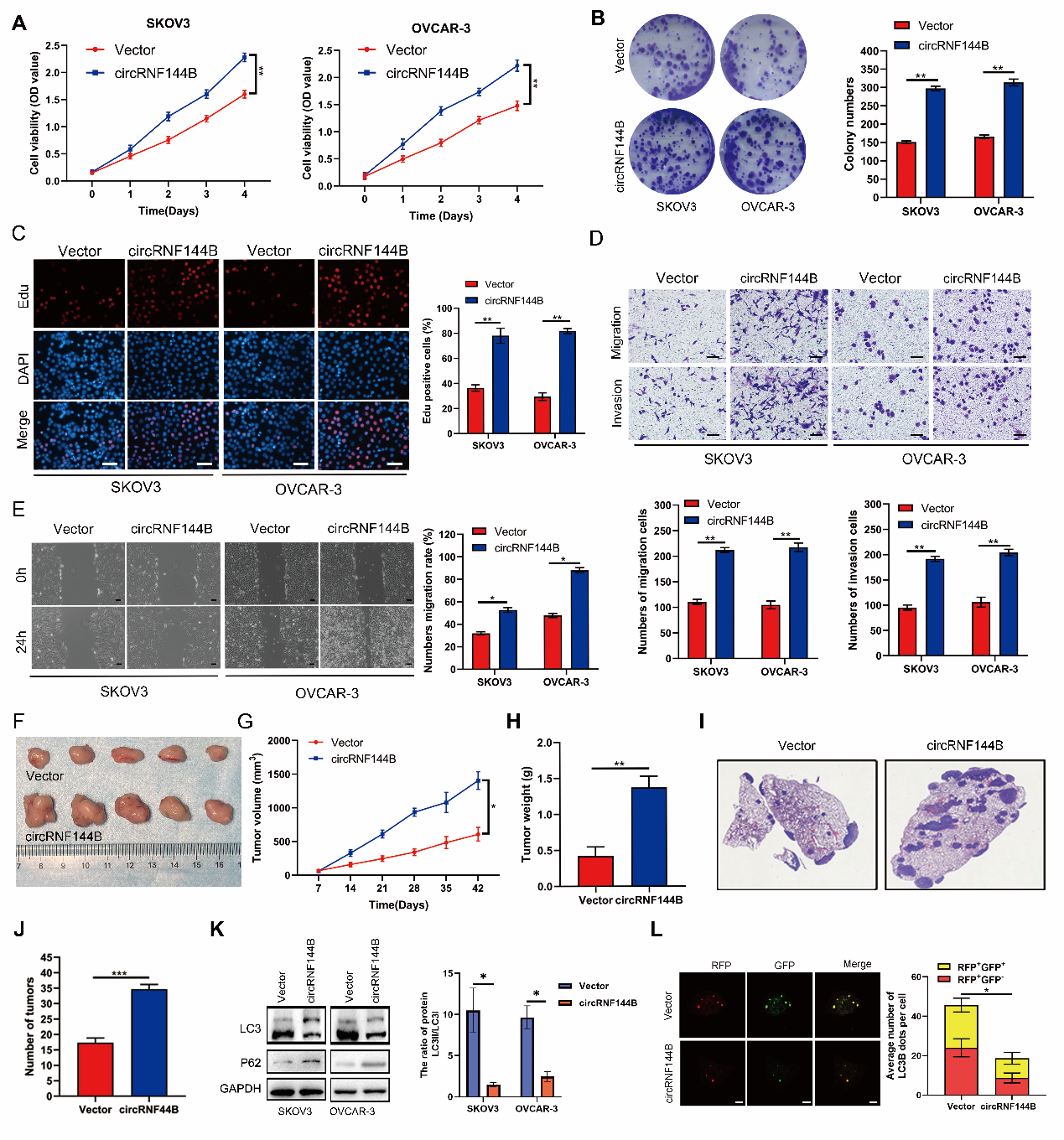


Figure S4 Confocal microscopy was used to detect the number of autophagosomes and autophagolysosomes in OVCAR-3 cells after circRNF144B overexpressed.


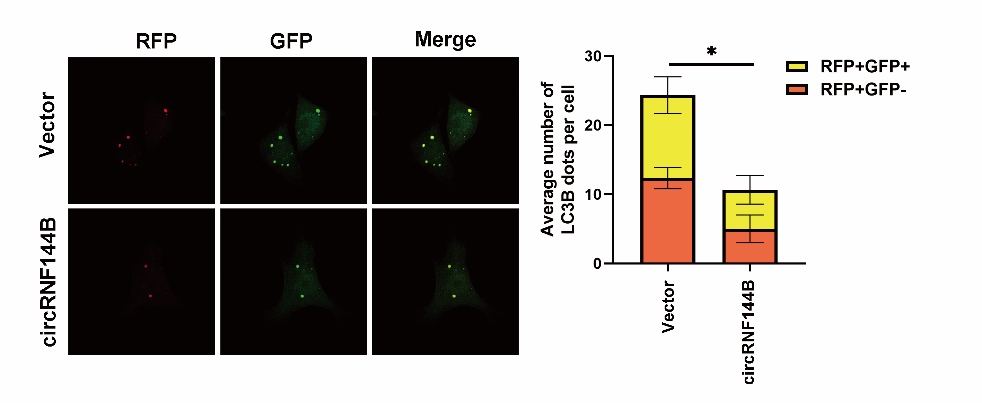


Figure S5 q-RT-PCR experiment verified the transfection efficiency of siRNA on FBXL11 in SKOV3 and OVCAR-3.


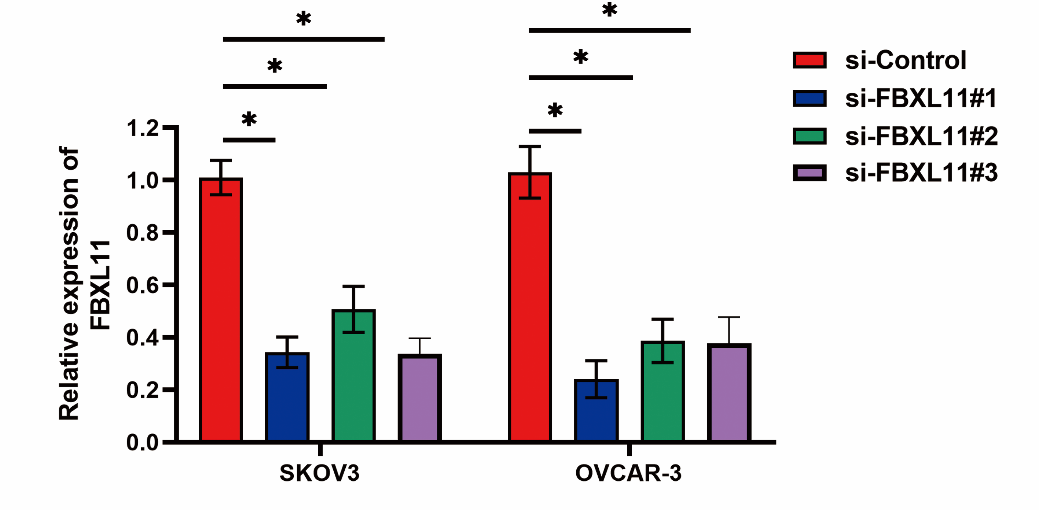


Figure S6 Confocal microscopy was used to detect the number of autophagosomes and autophagolysosomes in each group of OVCAR-3.


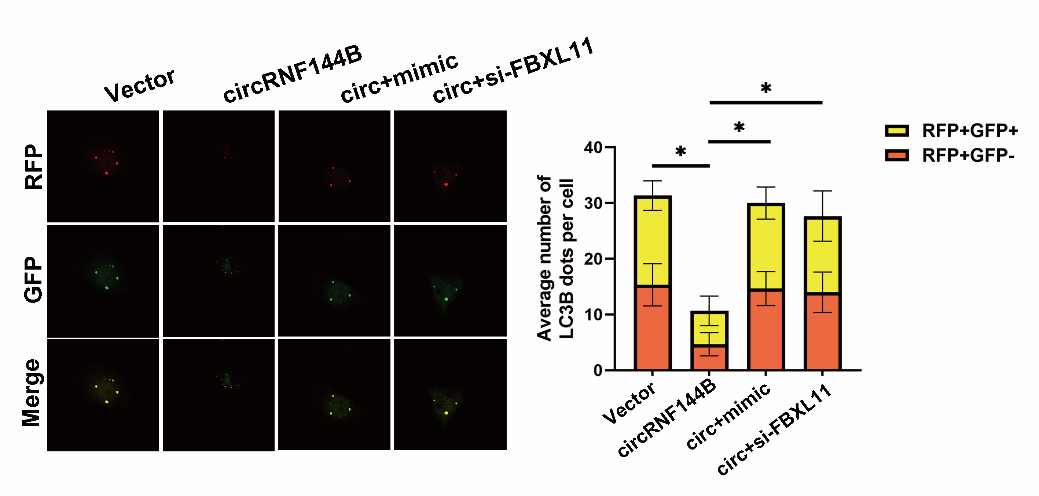


Figure S7 The secondary mass spectrograms indicated these proteins（A. CREBBP, B. ATN1, C.KIF20B ）binding with FBXL11.


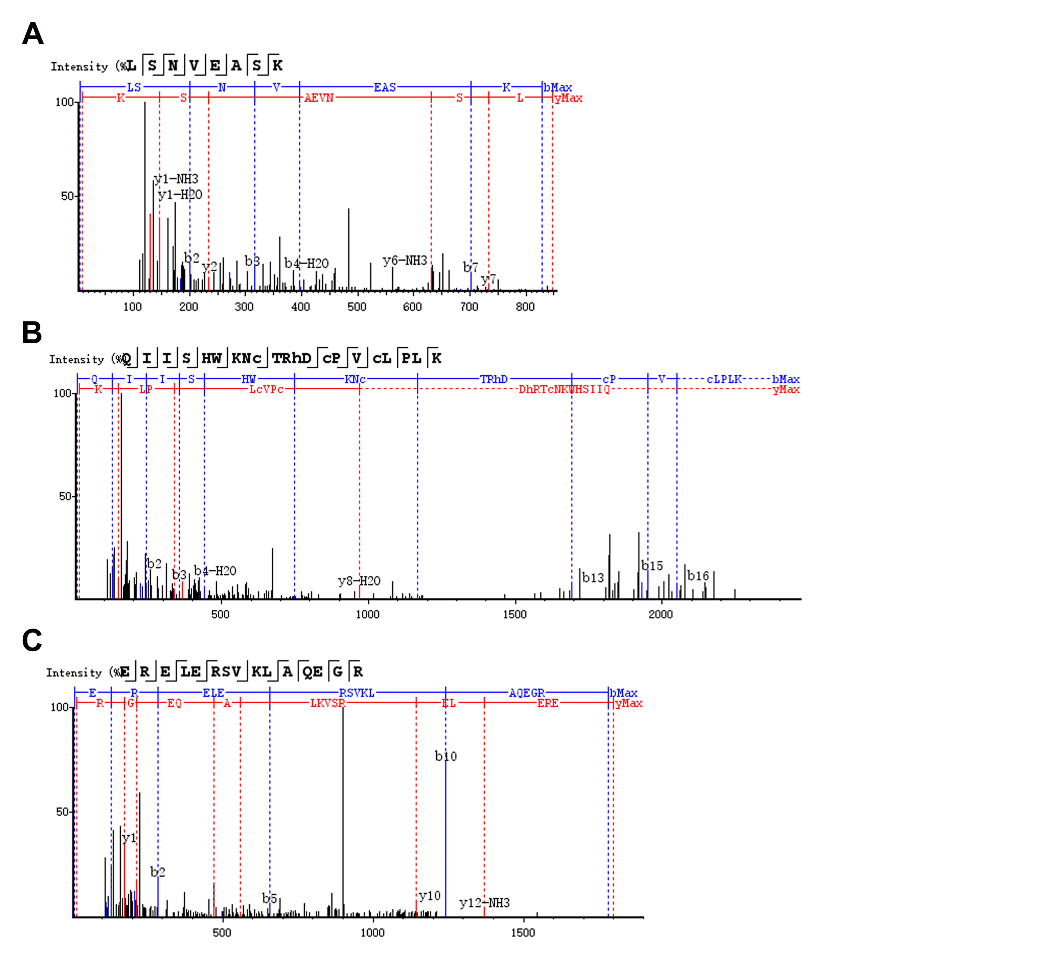


Figure S8 Confocal microscopy was used to detect the number of autophagosomes and autophagolysosomes in each group of OVCAR-3.


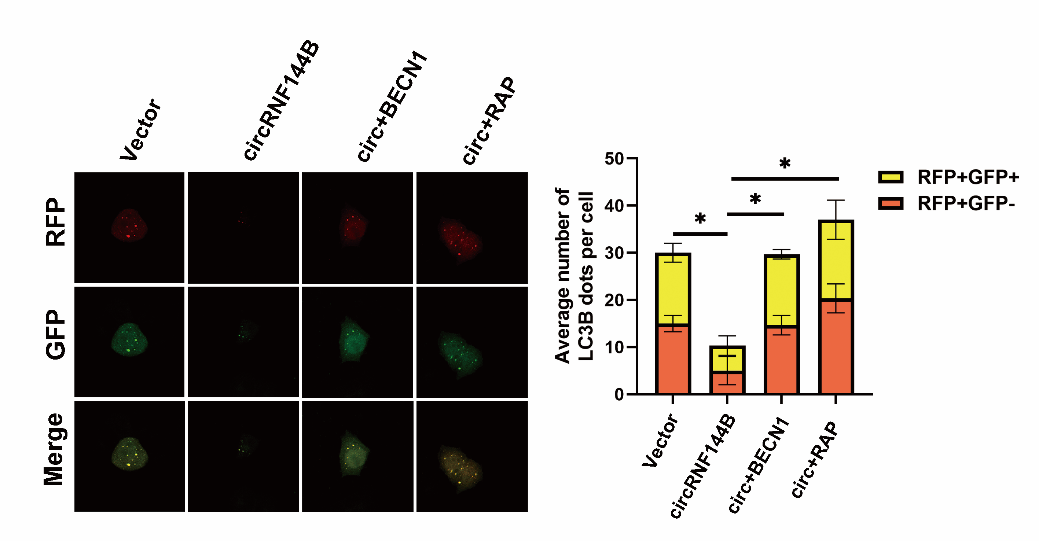

Supplement: Supplementary file 3 — Supplementary Figure [file 41419_2022_5286_MOESM3_ESM.docx]
