# Supplementary material for: CircRNF144B/miR-342-3p/FBXL11 axis reduced autophagy and promoted the progression of ovarian cancer by increasing the ubiquitination of Beclin-1
Source: Cell Death Dis. 2022 Oct 8;13(10):857. doi: 10.1038/s41419-022-05286-7 (PMC9547922; doi:10.1038/s41419-022-05286-7)

FIG.3K

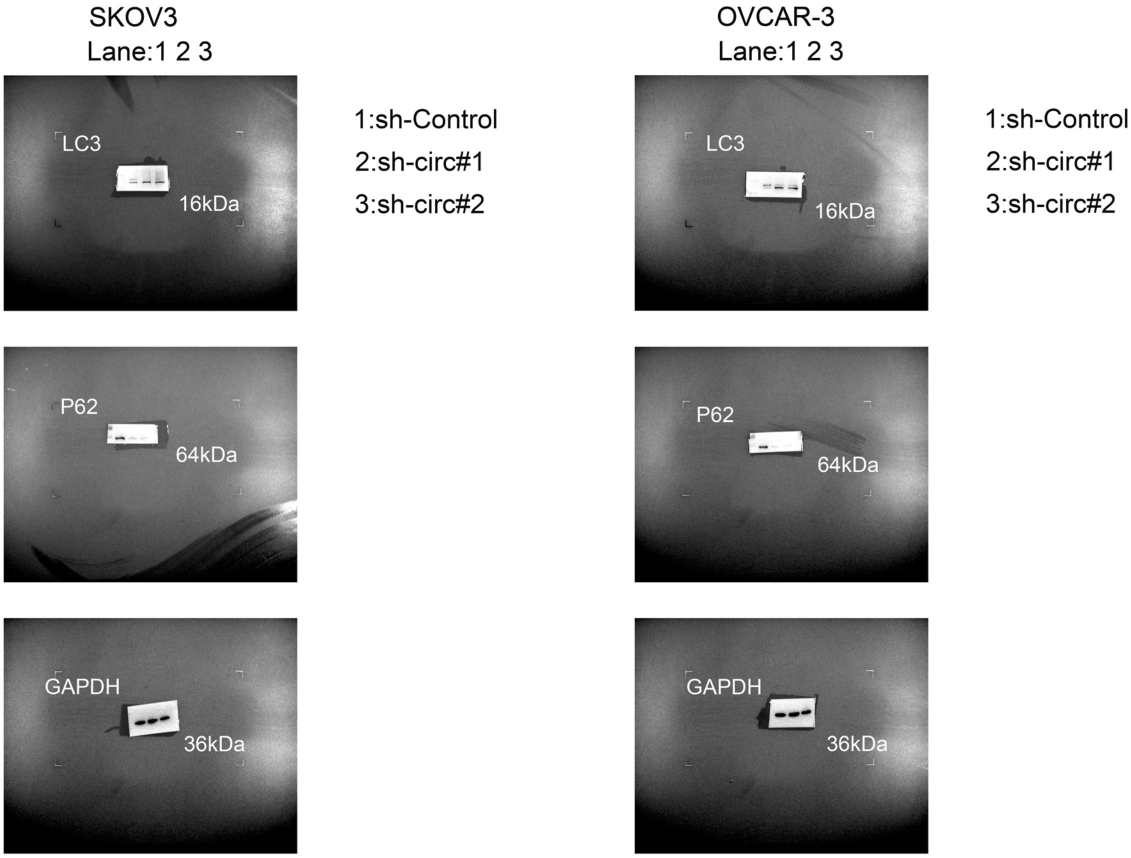

FIG.S3K

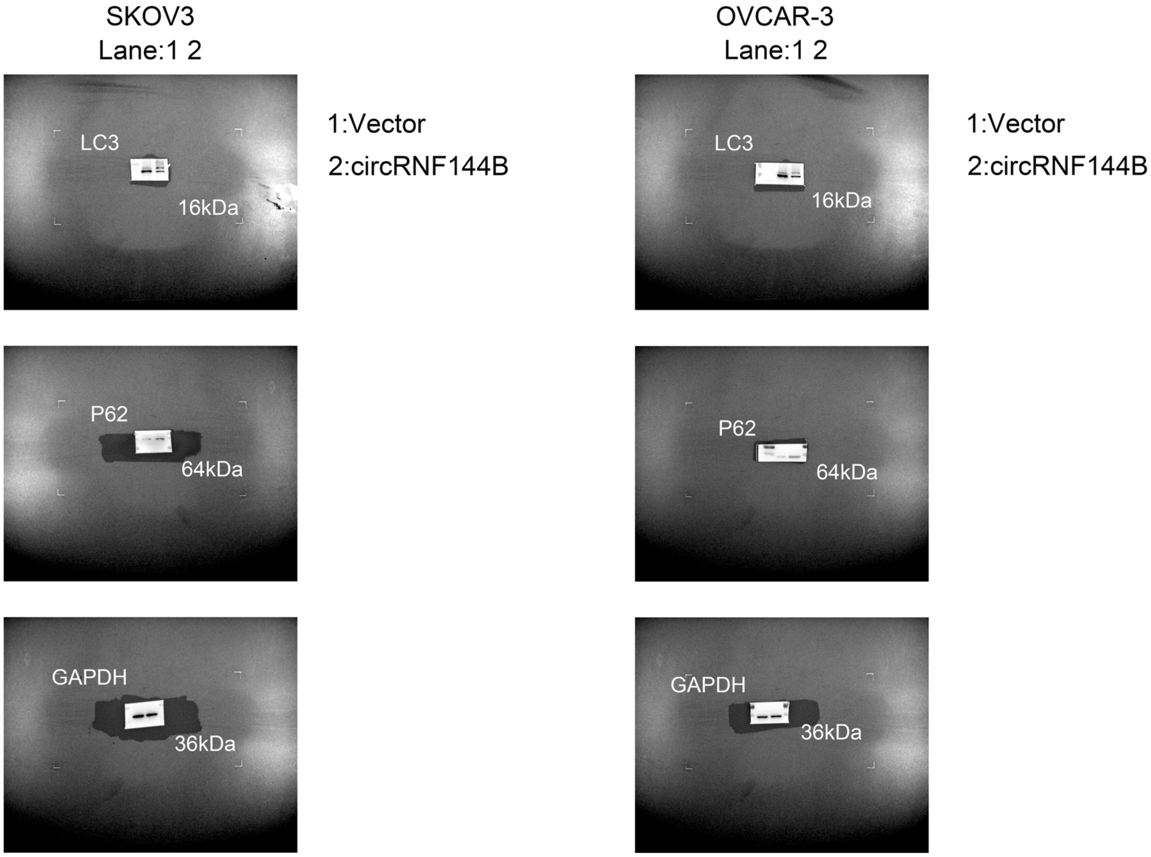

FIG.5E SKOV3

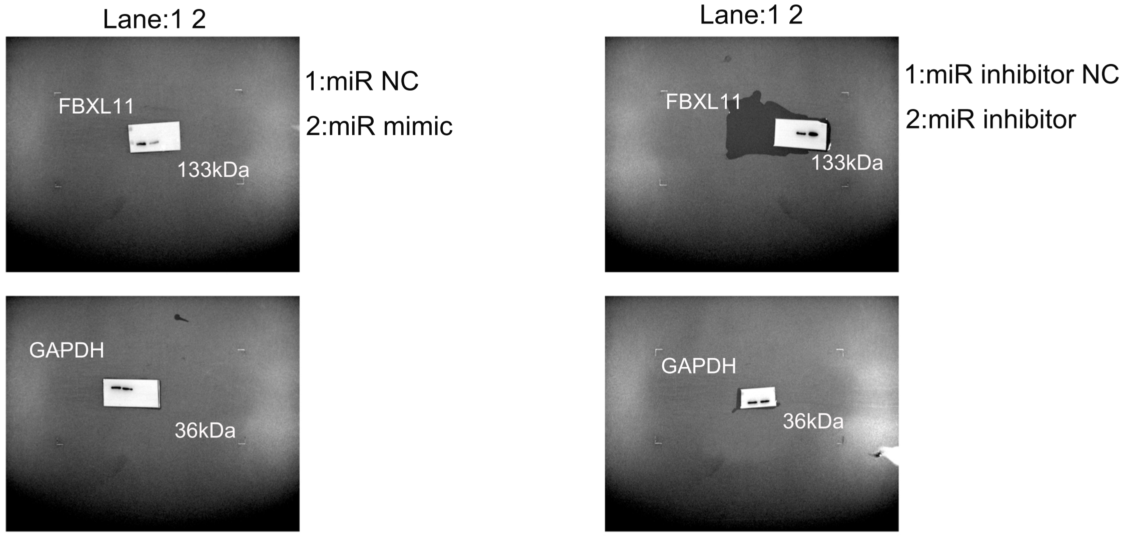

FIG.5E OVCAR-3

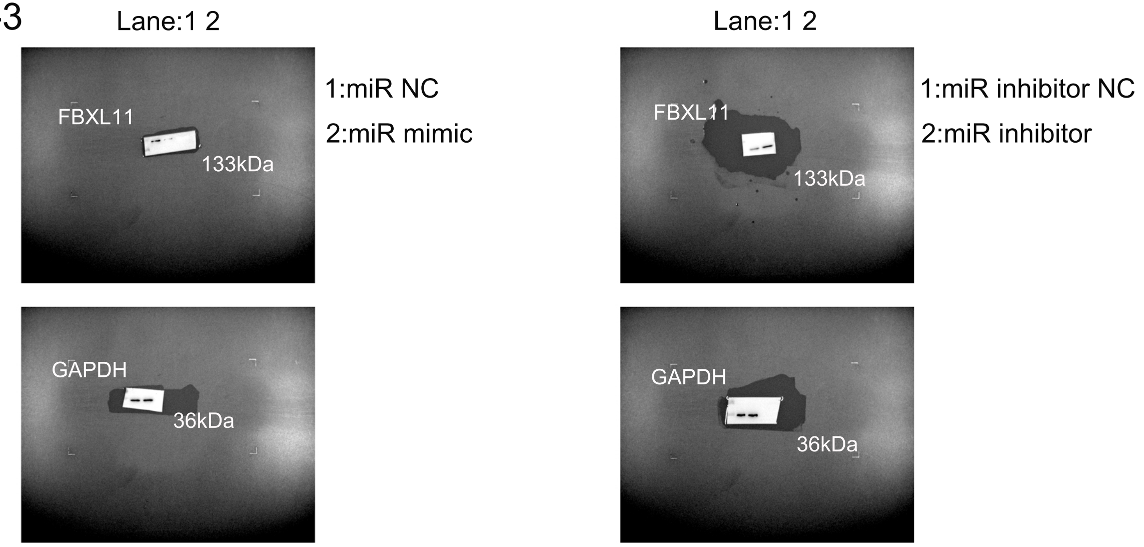

FIG.6F

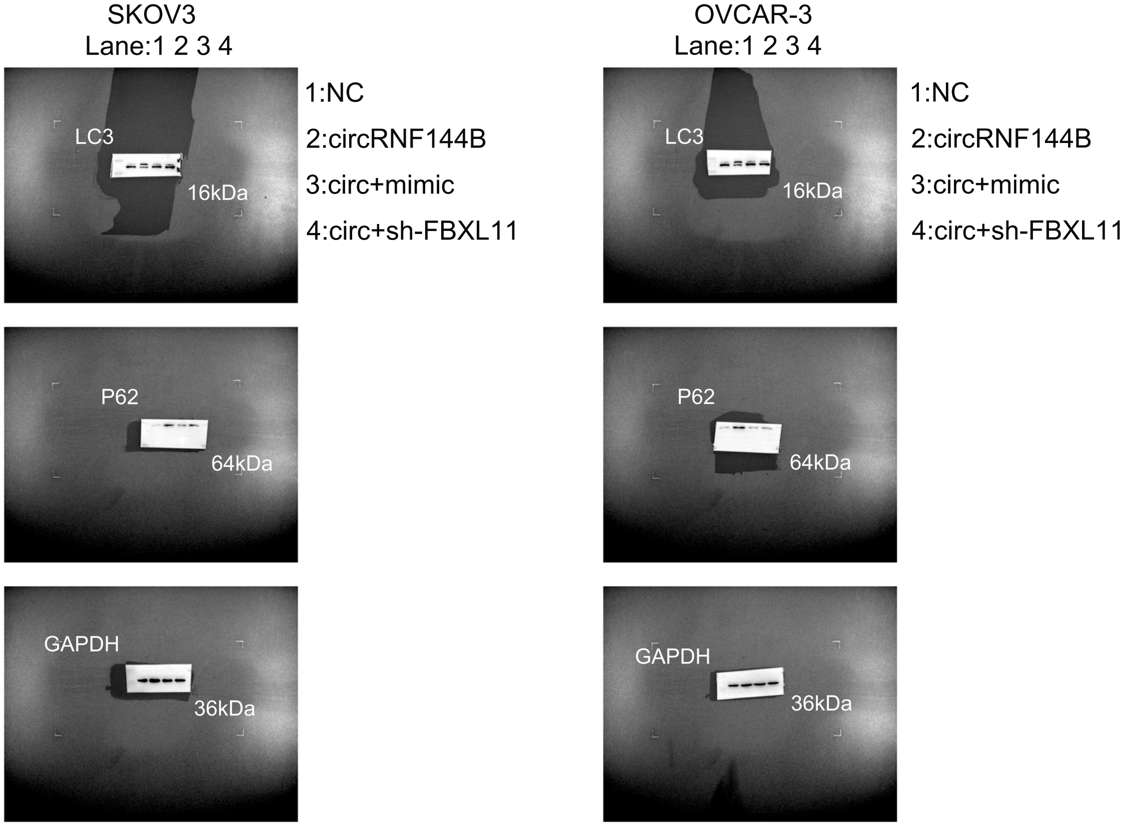

FIG.7C

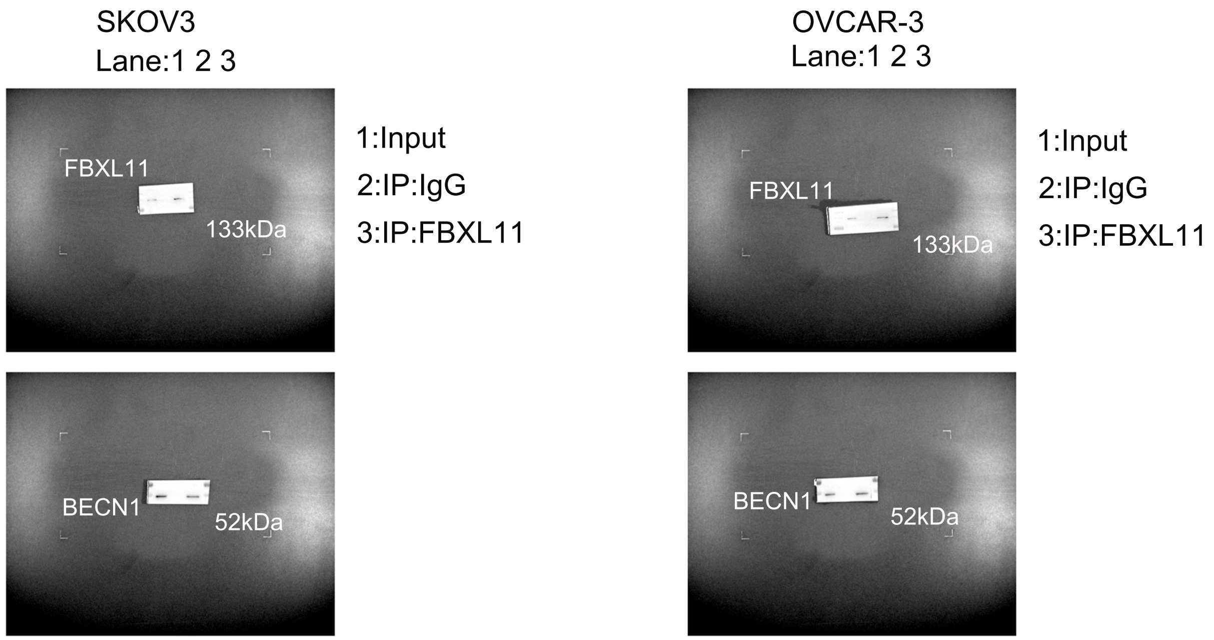
SKOV3
Lane:1 2 3
FBXL11
133kDa
BECN1
52kDa
1:Input
2:IP:IgG
3:IP:FBXL11
OVCAR-3
Lane:1 2 3
FBXL11
133kDa
BECN1
52kDa
1:Input
2:IP:IgG
3:IP:FBXL11

FIG.7D

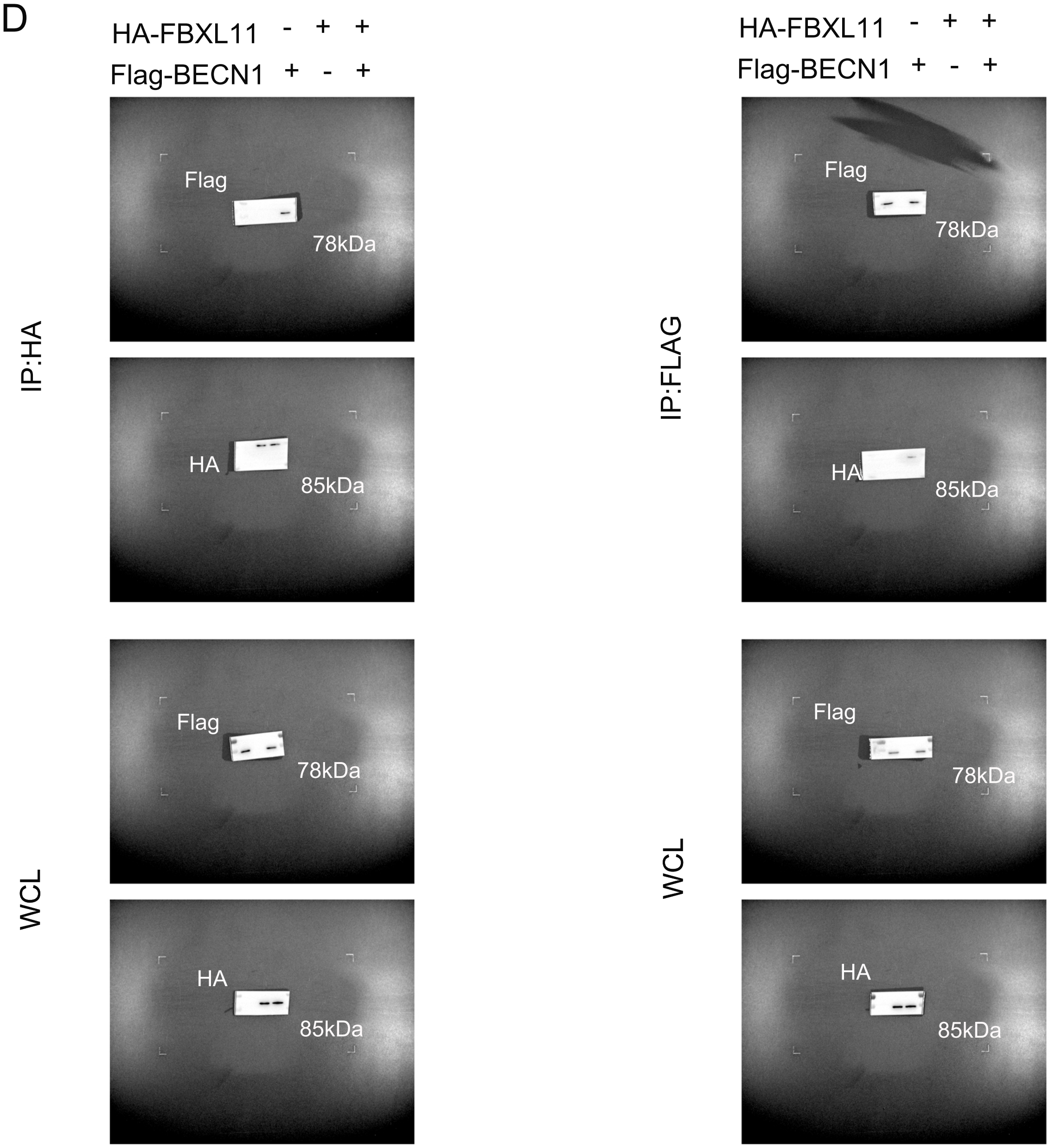
HA-FBXL11 - + +
Flag-BECN1 + - +
IP:HA
Flag
78kDa
HA
85kDa
WCL
Flag
78kDa
HA
85kDa
HA-FBXL11 - + +
Flag-BECN1 + - +
IP:FLAG
Flag
78kDa
HA
85kDa
WCL
Flag
78kDa
HA
85kDa

FIG.7E

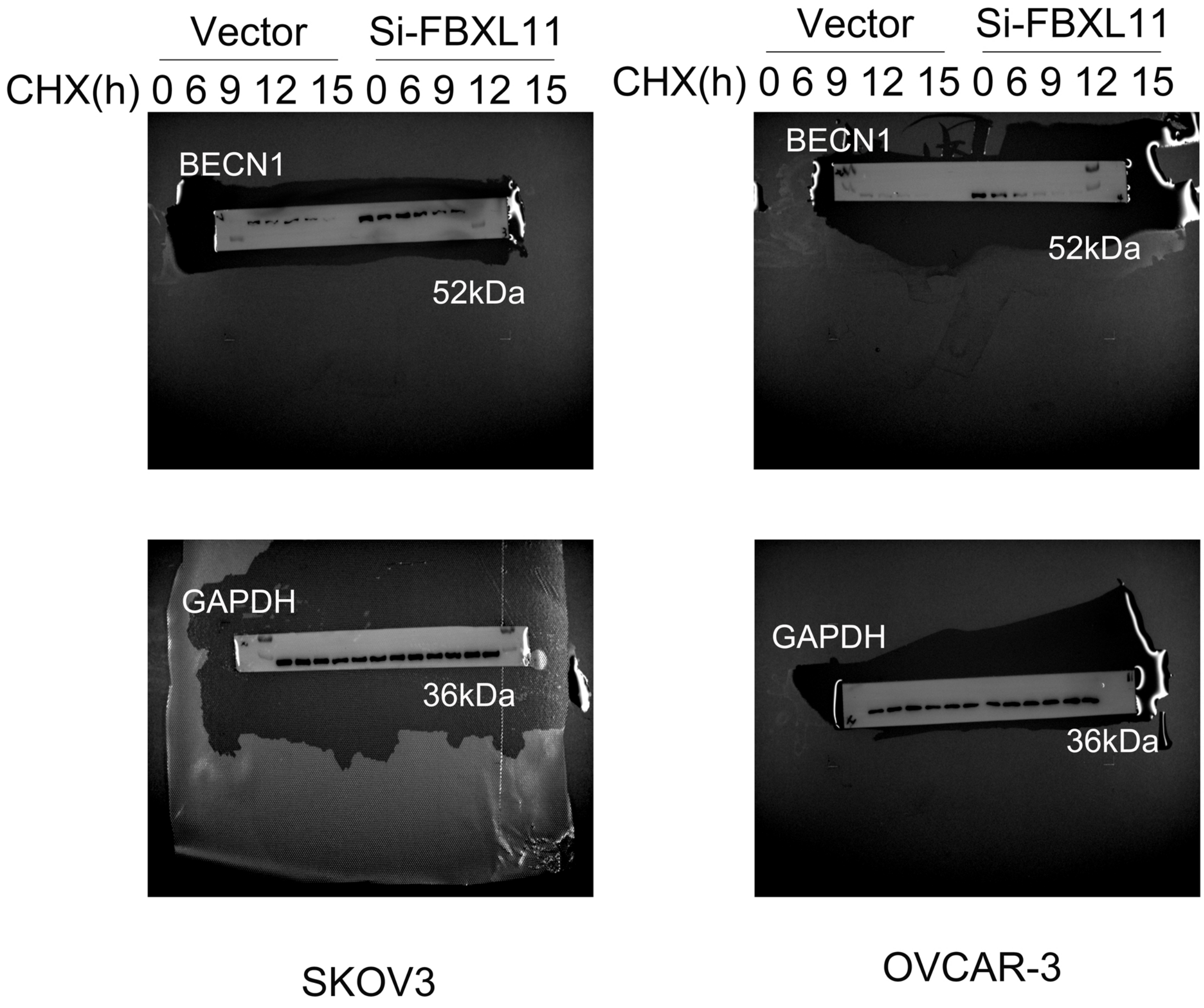
Vector
Si-FBXL11
CHX(h) 0 6 9 12 15 0 6 9 12 15
BECN1
52kDa
GAPDH
36kDa
Vector
Si-FBXL11
CHX(h) 0 6 9 12 15 0 6 9 12 15
BECN1
52kDa
GAPDH
36kDa
SKOV3
OVCAR-3

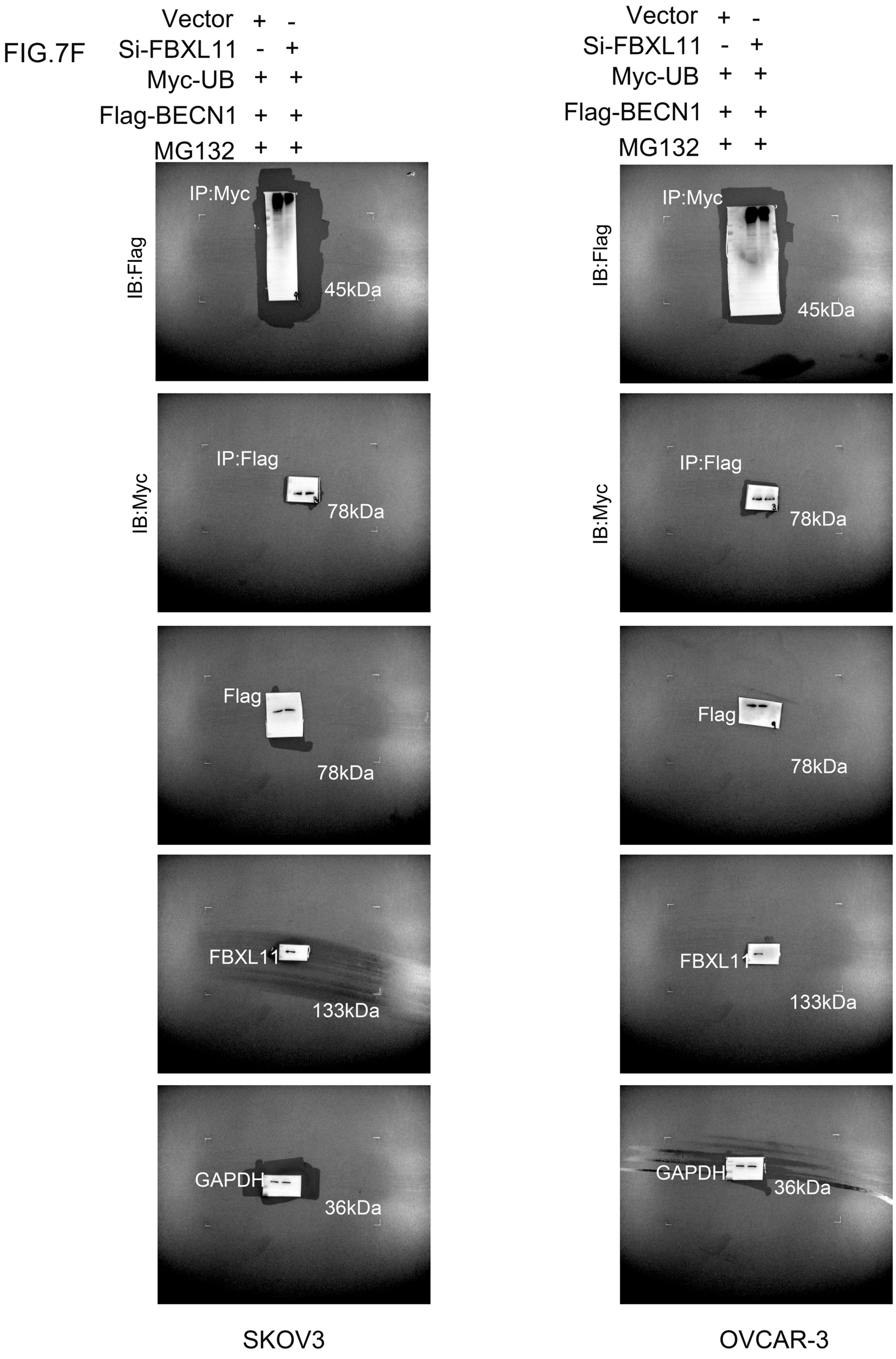
FIG.7F
Vector + -
Si-FBXL11 - +
Myc-UB + +
Flag-BECN1 + +
MG132 + +
IB:Flag
IP:Myc
45kDa
IB:Myc
IP:Flag
78kDa
Flag
78kDa
FBXL11
133kDa
GAPDH
36kDa
SKOV3
OVCAR-3

FIG.8A

SKOV3
Lane:1 2 3 4

OVCAR-3
Lane:1 2 3 4

1:NC
2:circRNF144B
3:circ+BECN1
4:circ+RAP

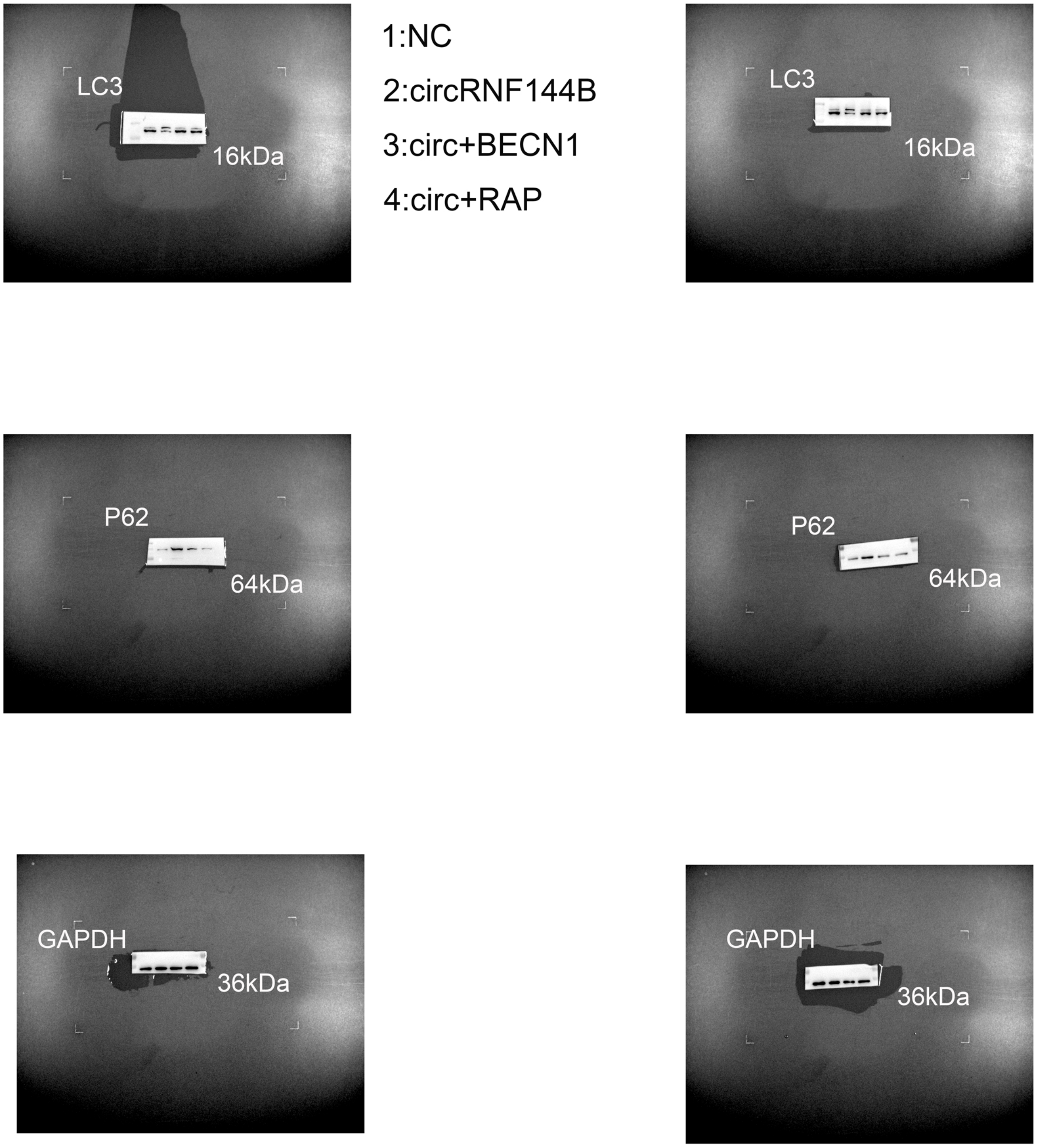

Supplement: Supplementary file 4 — Original Data File [file 41419_2022_5286_MOESM4_ESM.pdf]
